# Acquisition of radioresistance in docetaxel-resistant human lung adenocarcinoma cells is linked with dysregulation of miR-451/c-Myc-survivin/rad-51 signaling

**DOI:** 10.18632/oncotarget.2176

**Published:** 2014-07-07

**Authors:** Rui Wang, Dong-Qin Chen, Jia-Yuan Huang, Kai Zhang, Bing Feng, Ban-Zhou Pan, Jing Chen, Wei De, Long-Bang Chen

**Affiliations:** ^1^ Department of Medical Oncology, Jinling Hospital, School of Medicine, Nanjing University, Nanjing, Jiangsu 210002, China; ^2^ Department of Biochemistry and Molecular Biology, Nanjing Medical University, Nanjing, Jiangsu 210002, China

**Keywords:** lung adenocarcinoma, chemoresistance, radioresistance, miR-451, c-Myc, rad-51, survivin

## Abstract

Chemoresistant tumors usually fail to respond to radiotherapy. However, the mechanisms involved in chemo- and radiotherapy cross resistance are not fully understood. Previously, we have identified microRNA (miR)-451 as a tumor suppressor in lung adenocarcinoma (LAD). However, whether miR-451 plays critical roles in chemo- and radiotherapy cross resistance in LAD is unclear. Here, we established two docetaxel-resistant LAD cell models (SPC-A1/DTX and H1299/DTX), and showed that miR-451 was significantly downregulated in docetaxel-resistant LAD cells. Gain - and loss - of - function assays indicated that re-expression of miR-451 could reverse radioresistance of docetaxel-resistant LAD cells both *in vitro* and *in vivo* through promoting apoptosis and DNA double-strand breaks (DSBs). The proto-oncogene c-Myc was identified as a direct target of miR-451, and re-expression of miR-451 inhibited survivin and rad-51 expression by reducing the amount of c-Myc protein binding to their promoters. Silencing of c-Myc could phenocopy the effects of miR-451 upregulation, and restoration of c-Myc could partially rescue the effect of miR-451 upregulation on radiosensitivity of docetaxel-resistant LAD cells. Therefore, dysregulation of miR-451/c-Myc-survivin/rad-51 signaling is responsible for radioresistance of docetaxel-resistant LAD cells, and targeting it will be a potential strategy for reversing chemo- and radiotherapy cross resistance of LAD patients.

## INTRODUCTION

LAD, the most common histological type of lung cancer, has been regarded as the leading cause of cancer-related deaths for both males and females worldwide and is always diagnosed at advanced stage [1]. The current therapies for advanced LAD mainly include surgery, radiation therapy, chemotherapy, local treatments and targeted therapies [2]. Chemotherapy combined with adjuvant radiotherapy has been utilized to improve the survival and prognosis of patients with advanced LAD. However, in clinic, chemoresistant LAD patients often fail to respond to other cytotoxic treatments including radiotherapy, which leads to poor prognosis of the patients [3]. However, the molecular mechanisms involved in chemo- and radiotherapy cross resistance are not fully understood. Therefore, it is urgent to understand the molecular mechanisms of aberrant treatment responses to exploit the novel strategies for sensitizing chemoresistant LAD patients to radiotherapy.

MiRNAs, a group of small noncoding RNAs, post-transcriptionally regulate gene expression by complementary binding to the 3'-UTR of their target genes [4]. It is well known that miRNAs are involved in a wide range of human physiological and pathological processes, including tumorigensis [5, 6]. Increasing evidence has shown that dysregulation of miRNAs plays a critical role in LAD development. For example, increased miR-21 expression is reported to be associated with disease progression and survival in stage I lung adenocarcinoma, suggesting that expression of miR-21 may contribute to lung carcinogenesis and serve as a therapeutic target or early-stage prognostic biomarker for lung adenocarcinoma [7]. In contrast, miR-145 and miR-182 are reported to be upregulated in LAD tissues. Specific miRNAs for early diagnosing or predicting the prognosis of LAD patients and their correlation between miRNA expression in tissues, serums and sputums are increasingly reported [8]. In addition, some miRNAs are reported to play critical roles in chemo- or radioresistance of LAD cells. For example, we previously identified a differential miRNA-expression profile between docetaxel-resistant and parental LAD cells, and showed that dysregulation of some miRNAs (miR-650, Let-7c, miR-200b and miR-100) was linked with the chemoresistance of LAD cells [9-12]. Also, overexpression of miR-449a in lung adenocarcinoma cell line (CL1-0) effectively increased irradiation-induced DNA damage and apoptosis, altered the cell cycle distribution and eventually led to sensitization of CL1-0 to irradiation [13]. Zhang and his colleagues showed that miR-511 could regulate the growth of radioresistant A549/R cells by increasing BAX expression through TRIB2 [14]. These data suggest that miRNA replacement therapy or anti-miRNA oligonucleotides will possess significant potentials for molecular targeted therapy of human malignancies. In our previous study, miR-451 was observed to be downregulated in non-small cell lung cancer and re-expression of miR-451 could inhibit growth and induce apoptosis in both LAD cells and squamous cell carcinoma (SCC) cells [15]. However, the roles of miR-451 in chemo- and radiotherapy cross resistance of LAD cells are not better understood and need to be further elucidated.

In the present study, we were the first to show that re-expression of miR-451 could significantly reverse the radioresistance of docetaxel-resistant LAD cells through promoting apoptosis and DNA DSBs both *in vitro* and *in vivo*. The proto-oncogene c-Myc, a key transcriptional factor for survivin and rad-51, was identified as a direct and functional target of miR-451. Thus, the newly identified miR-451/c-Myc-survivin/rad-51 signaling was linked with chemo- and radiotherapy cross resistance of human LAD cells.

## RESULTS

### Docetaxel-resistant LAD cells is cross-resistant to radiation

To develop an *in vitro* model of acquired docetaxel resistance in LAD, docetaxel-resistant SPC-A1 cell line (SPC-A1/DTX) and H1299 cell line (H1299/DTX) were previously established in our lab [16, 17]. Specifically, to establish docetaxel-resistant LAD cells, parental LAD cells were continuously exposed to docetaxel for more than 1 year until cells had acquired resistance to docetaxel. Results from MTT assay indicated that the IC_50_ values to docetaxel in SPC-A1/DTX or H1299/DTX cell lines (605.44±46.3 μg/L or 587.83±33.4 μg/L) were significantly higher than those in parental SPC-A1 or H1299 cell lines (123.69±10.3 μg/L or 170.15±15.14 μg/L) (Supplementary Figure 1A), suggesting that SPC-A1/DTX or H1299/DTX cells had indeed acquired docetaxel resistance. To further explore whether docetaxel-resistant LAD cells is cross-resistant to radiation, we determined the 50% effective dose (ED_50_) values of irradiation in docetaxel-resistant and parental LAD cells. Results from Cell Counting Kit-8 (CCK-8) assay indicated that the ED50 values of irradiation in SPC-A1/DTX or H1299/DTX cell lines (12.2±1.2 Gy or 11.1±0.9 Gy) were significantly higher than those in parental SPC-A1 or H1299 cell lines (3.4±0.5 Gy and 3.1±0.3 Gy) (Supplementary Figure 1B). Colony formation assays also showed significant radioresistance in SPC-A1/DTX and H1299/DTX cells compared with parental SPC-A1 and H1299 cells (Supplementary Figure 1C). To investigate whether cross-resistance to irradiation was correlated with irradiation-induced apoptosis and DNA DSBs, flow cytometry was performed to detect the changes of apoptosis and Western blotting was performed to detect the phosphorylation expression of H2A.X (γ-H2A.X) protein, which was identified as a marker of DNA DSBs. In docetaxel-resistant SPC-A1/DTX and H1299/DTX cell lines, there was a significant decrease in apoptosis on exposure to various doses of irradiation in comparison with parental SPC-A1 and H1299 cell lines (Supplementary Figure 1D). Also, the expression level of γ-H2A.X protein in SPC-A1/DTX or H1299/DTX cells was significantly lower than that in parental SPC-A1 or H1299 cells (Supplementary Figure 1E). Therefore, abrogation of apoptosis and the decreased phosphorylation expression of H2A.X foci formation might be involved in chemo- and radiotherapy cross resistance of LAD cells.

### Downregulation of miR-451 was correlated with radioresistance of docetaxel-resistant LAD cells

**Figure 1:**
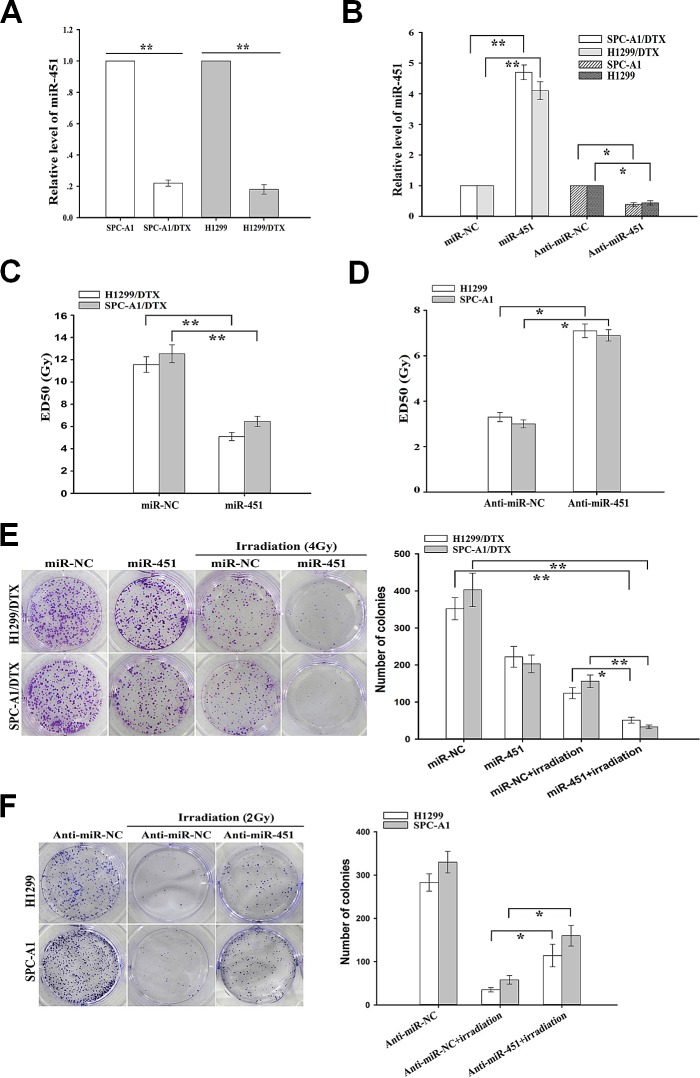
Effect of miR-451 expression on *in vitro* radiosensitivity of docetaxel-resistant LAD cells (A) qRT-PCR detection of relative miR-451 expression in docetaxel-resistant LAD cells (SPC-A1/DTX and H1299/DTX) and their parental LAD cells (SPC-A1 and H1299). U6 was used as an internal control. (B) qRT-PCR detection of relative miR-451 expression in pcDNA/miR-451(or pcDNA/miR-NC)-transfected SPC-A1/DTX or H1299/DTX cells, and Anti-miR-451 (or Anti-miR-NC)-transfected SPC-A1 or H1299 cells, respectively. U6 was used as an internal control. (C) CCK-8 assay was conducted to detect the ED_50_ values of irradiation to pcDNA/miR-451(or pcDNA/miR-NC)-transfected SPC-A1/DTX or H1299/DTX cells. (D) CCK-8 assay was conducted to detect the ED_50_ values of irradiation to Anti-miR-451 (or Anti-miR-NC)-transfected SPC-A1 or H1299 cells. (E) The colony formation of pcDNA/miR-451(or pcDNA/miR-NC)-transfected SPC-A1/DTX or H1299/DTX cells treated without irradiation or with irradiation (4.0 Gy). (F) The colony formation of Anti-miR-451 (or Anti-miR-NC)-transfected SPC-A1 or H1299 cells treated without irradiation or with irradiation (2.0 Gy). Results represent the average of three independent experiments (mean±SD). *P<0.05 and **P< 0.01.

Our previous study has shown that miR-451 functions as a potent tumor suppressor in human NSCLC [15], but the roles of miR-451 in chemo- and radiotherapy cross resistance of LAD are sill unclear. qRT-PCR was performed to detect the expression of miR-451 in docetaxel-resistant and parental LAD cells, and results indicated that miR-451 was significantly downregulated in SPC-A1/DTX and H1299/DTX cells in comparison with the corresponding parental SPC-A1 and H1299 cells (Figure 1A). To further understand the effect of miR-451 expression on the radiosensitivity of docetaxel-resistant LAD cells, pcDNA/miR-451 (or pcDNA/miR-NC) or Anti-miR-451 (or Anti-miR-NC) was stably or transiently transfected into docetaxel-resistant or parental LAD cells, respectively. The results of qRT-PCR confirmed the upregulation of miR-451 in pcDNA/miR-451-transfected SPC-A1/DTX or H1299/DTX cells and the downregulation of miR-451 in Anti-miR-451-transfected SPC-A1 or H1299 cells, in comparison with respective control cells (Figure 1B). Then, the effect of miR-451 expression on radiosensitivity of LAD cells was determined by the clonogenic survival assay. The ED_50_ values for irradiation in miR-451-transfected SPC-A1/DTX or H1299/DTX cells were significantly decreased by 48.0% or 56.1%, respectively, in comparison with those in miR-NC-transfected cells (Figure 1C). Meanwhile, the ED_50_ values for irradiation in Anti-miR-451-transfected SPC-A1 or H1299 cells were significantly increased by 30.0% or 15.2%, respectively, in comparison with those in Anti-miR-NC-transfected cells (Figure 1D). Compared with that of miR-NC-transfected cells combined with irradiation treatment (4.0Gy), the capacity of colony formation was significantly decreased in miR-451-transfected H1299/DTX or SPC-A1/DTX cells combined with irradiation treatment (4.0Gy) (Figure 1E). Compared with that of miR-NC-transfected cells combined with irradiation treatment, the capacity of colony formation was significantly increased in anti-miR-451-transfected H1299/DTX or SPC-A1/DTX cells combined with irradiation treatment (2.0Gy) (Figure 1F). These data indicated that miR-451 repression might play critical roles in radioresistance of docetaxel-resistant LAD cells.

### Re-expression of miR-451 significantly increases irradiation-induced apoptosis and DNA DSBs of docetaxel-resistant LAD cells

**Figure 2:**
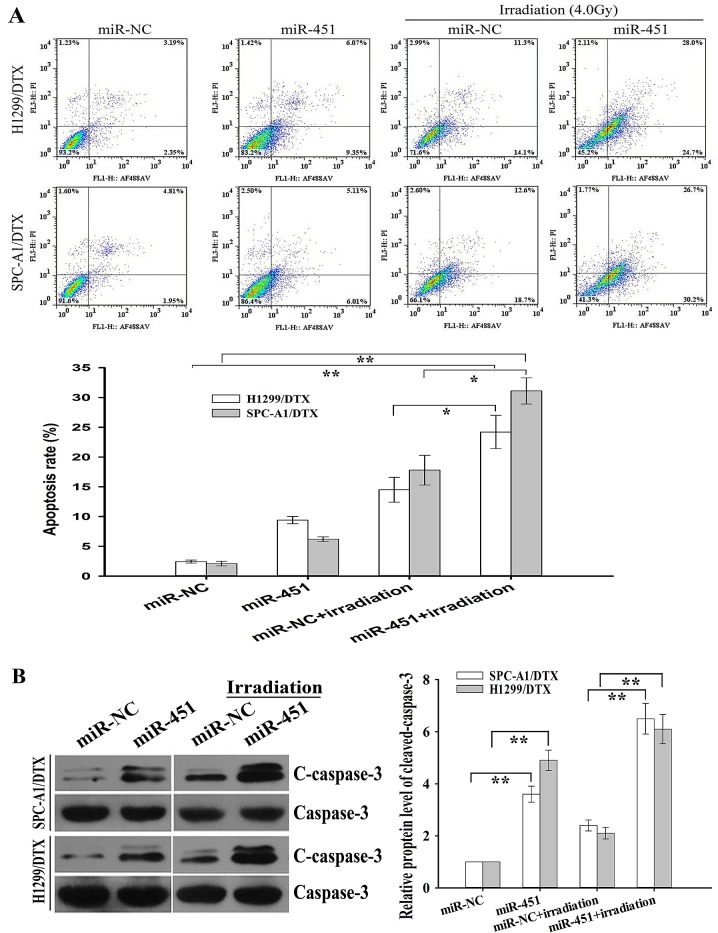
Effect of miR-451 expression on irradiation-induced apoptosis of docetaxel-resistant LAD cells (A) Flow cytometric analysis of apoptosis in pcDNA/miR-451(or pcDNA/miR-NC)-transfected SPC-A1/DTX or H1299/DTX treated without irradiation or with irradiation (4.0 Gy). (B) Western blotting detection of cleaved Caspase-3 (C-caspase-3) and total Caspase-3 (Caspase-3) in pcDNA/miR-451(or pcDNA/miR-NC)-transfected SPC-A1/DTX or H1299/DTX treated without irradiation or with irradiation (4.0 Gy). GAPDH was used as an internal control. Results represent the average of three independent experiments (mean±SD). *P< 0.05 and **P< 0.01.

Next, we focused on revealing the underlying molecular mechanisms by which re-expression of miR-451 reversed radioresistance of docetaxel-resistant LAD cells. Apoptotic cell death has already been associated with radioresistance of several tumor cells [18]. Flow cytometry was performed to determine the effect of miR-451 expression on irradiation-induced apoptosis in docetaxel-resistant and parental LAD cells, and it was observed that the apoptosis of miR-451-enforced H1299/DTX or SPC-A1/DTX cells combined with irradiation treatment (4.0 Gy) was significantly increased compared with that in miR-NC-transfected cells combined with irradiation treatment (Figure 2A). Likewise, when combined with irradiation treatment (2.0Gy), the apoptosis of Anti-miR-451-transfected parental H1299 or SPC-A1 cells was significantly decreased compared with Anti-miR-NC-transfected cells (Supplementary Figure 2A). Next, Western blot assay was performed to detect the expression of total Caspase-3 (Caspase-3) and Cleaved caspase-3 (C-caspase-3). Compared with those cells transfected with miR-NC alone or combined with irradiation treatment (4.0 Gy), the expression of C-caspase-3 protein in miR-451-enforced H1299/DTX and SPC-A1/DTX cells combined with irradiation treatment was significantly increased, but the expression of total caspase-3 protein showed no significant difference (Figure 2B). Meanwhile, compared with those cells transfected with Anti-miR-NC alone or combined with irradiation treatment (2.0 Gy), the expression of C-caspase-3 protein in miR-451-downregulated H1299 and SPC-A1 cells combined with irradiation treatment was significantly decreased, but the expression of total Caspase-3 protein showed no significant difference (Supplementary Figure 2B). Collectively, upregulation of miR-451 could lead to the increased irradiation-induced apoptosis in docetaxel-resistant LAD cells.

**Figure 3:**
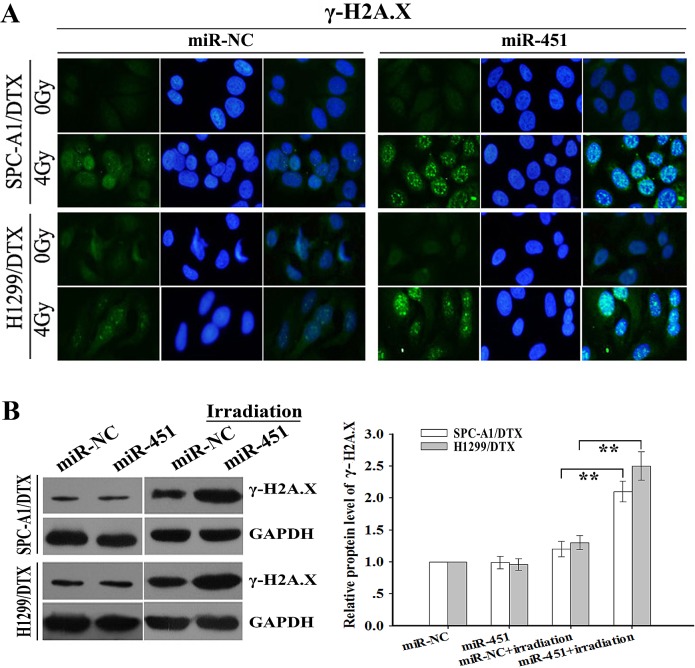
Effect of miR-451 expression on irradiation-mediated DNA DSBs of docetaxel-resistant LAD cells (A) Immunofluorescence detection of phosphorylation of H2A.X (γ-H2A.X) foci formation (a marker of DSB) in pcDNA/miR-451(or pcDNA/miR-NC)-transfected SPC-A1/DTX or H1299/DTX treated without irradiation or with irradiation (4.0 Gy). (B) Western blotting detection of γ-H2A.X protein expression in pcDNA/miR-451(or pcDNA/miR-NC)-transfected SPC-A1/DTX or H1299/DTX treated without irradiation or with irradiation (4.0 Gy). GAPDH was used as an internal control. Results represent the average of three independent experiments (mean±SD). **P< 0.01.

Phosphorylation of H2A.X (γ-H2A.X) foci formation, a marker of DNA DSBs, has been implicated in the process of DNA impairment induced by irradiation [18]. To further study the effect of miR-451 on regulation of irradiation-induced DNA DSBs, pcDNA/miR-451 or pcDNA/miR-NC was stably transfected into docetaxel-resistant LAD cells, and then the cells were treated with irradiation treatment (4.0 Gy). Then, immunofluorescence analysis of γ-H2A.X foci formation and Western blotting analysis of γ-H2A.X protein expression were performed. As shown in Figure 3A, when combined with irradiation treatment (4.0 Gy), miR-451 overexpression significantly increased γ-H2A.X foci formation compared with miR-NC group, and Western blotting analysis indicated that the expression level of γ-H2A.X protein was significantly upregulated in pcDNA/miR-451-transfected cells compared with in miR-NC-transfected group (Figure 3B). At the same time, when combined with irradiation treatment (2.0 Gy), the expression level of γ-H2A.X protein was significantly downregulated in Anti-miR-451-transfected cells compared with that in Anti-miR-NC-transfected group (Supplementary Figure 2C). Thus, re-expression of miR-451 enhances irradiation-induced apoptosis and DNA DSBs in docetaxel-resistant LAD cells.

### Re-expression of miR-451 significantly increases *in vivo* radiosensitivity of docetaxel-resistant LAD cells

**Figure 4:**
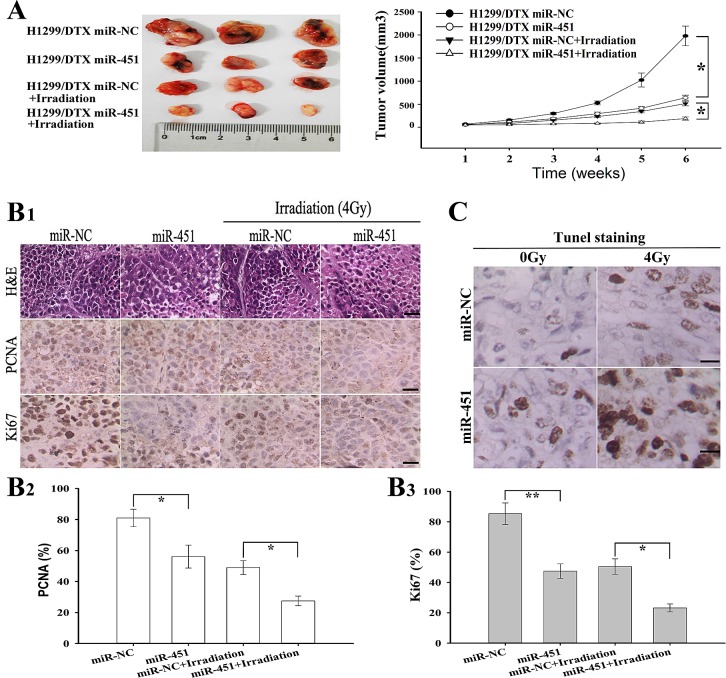
Effect of miR-451 expression on *in vivo* radiosensitivity of docetaxel-resistant LAD cells (A) Growth of tumors in the nude mice subcutaneously transplanted with 3.0×10^6^ pcDNA/miR-NC or pcDNA/miR-451-transfected H1299/DTX cells combined with irradiation treatment. Six mice of each group were used for the subcutaneous transplantation. Representative photographs of tumors were provided at 6 weeks after the inoculation. Data were the mean ± standard error. (B1-3), Hematoxylin and eosin (H & E) staining analysis of proliferating cell nuclear antigen (PCNA and Ki67) expression in tumors developed from pcDNA/miR-NC or pcDNA/miR-451-transfected H1299/DTX cells combined with irradition treatment. *Bars*, 50 μm. (C)TUNEL staining detection of apoptosis in tumors developed from pcDNA/miR-NC or pcDNA/miR-451-transfected H1299/DTX cells combined with irradiation treatment. *Bars*, 50μm. Results represent the average of three independent experiments (mean ± SD). *P< 0.05 and **P< 0.01.

To further investigate the effect of miR-451 expression on the *in vivo* radiosensitivity of LAD cells, H1299/DTX cells stably transfected pcDNA/miR-451 or pcDNA/miR-NC were subcutaneously inoculated into nude mice. After formation of palpable tumors, irradiation was performed and tumor volumes were measured. As shown in Figure 4A, the average volume of tumors developed from pcDNA/miR-451-transfected-H1299/DTX cells was significantly smaller compared with that developed from pcDNA/miR-NC-transfected cells, when combined with irradiation treatment. The tumors of H1299/DTX/miR-451 group grew more slowly than those of H1299/DTX/miR-NC group, when combined with irradiation treatment (Figure 4A). Then, immunostaining assay showed that the PCNA-positive and Ki67-positive cells in tumors derived from H1299/DTX/miR-451 group were significantly reduced than those in tumors derived from H1299/DTX/miR-NC group, when combined with irradiation treatment (Figure 4B1-3). Also, TUNEL staining assay indicated that the number of apoptotic cells in tumors derived from H1299/DTX/miR-451 group was significantly increased in comparison with those derived from H1299/DTX/miR-NC one, when combined with irradiation treatment (Figure 4C). Therefore, re-expression of miR-451 significantly increases the *in vivo* radiosensitivity of docetaxel-resistant LAD cells.

### Re-expression of miR-451 downregulates rad-51 and survivin expression by post-transcriptionally downregulating c-Myc

**Figure 5:**
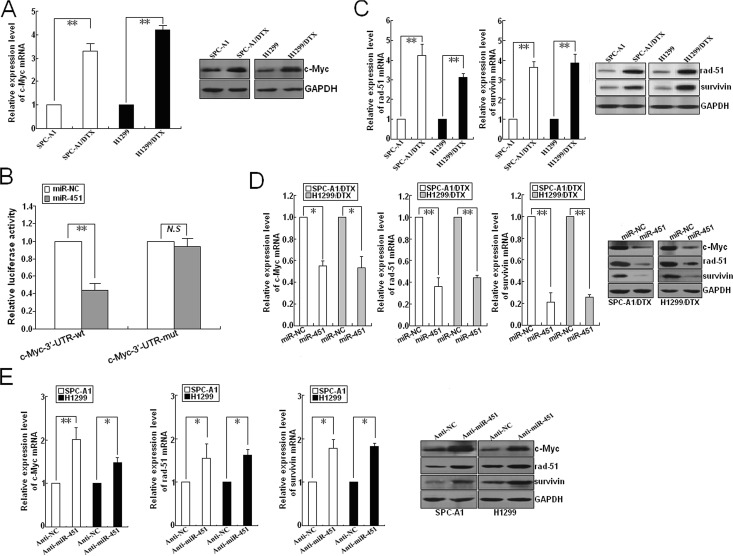
Identification of c-Myc as a target of miR-451 in LAD cells (A) qRT-PCR and Western blotting detection of c-Myc mRNA and protein expression in docetaxel-resistant LAD cells (SPC-A1/DTX and H1299/DTX) and parental LAD cells (SPC-A1 and H1299). (B) Relative luciferase activity was analyzed after wildtype (pLUC/c-Myc/3'-UTR-wt) or mutant (pLUC/c-Myc/3'-UTR-mut) 3'-UTR reporter plasmids were co-transfected with pcDNA/miR-NC or pcDNA/miR-451 in SPC-A1/DTX cells. The histogram shows the mean±SD of the normalized luciferase activity from three independent experiments. (C) qRT-PCR and Western blotting detection of rad-51 and survivin mRNA and protein expression in docetaxel-resistant and parental LAD cells. (D) Effect of miR-451 upregulation on c-Myc, rad-51 and survivin expresssion in docetaxel-resistant LAD cells. qRT-PCR and Western blotting assays were performed to detect the mRNA and protein expression of c-Myc, rad-51 and survivin in pcDNA/miR-NC or pcDNA/miR-451-transfected SPC-A1/DTX and H1299/DTX cells. (E) Effect of miR-451 downregulation on c-Myc, rad-51 and survivin expression in parental SPC-A1 and H1299 cells. qRT-PCR and Western blotting assays were performed to detect the mRNA and protein expression of c-Myc, rad-51 and survivin in Anti-miR-NC or Anti-miR-451-transfected SPC-A1 and H1299 cells. GAPDH was used as an internal control. Results represent the average of three independent experiments (mean±SD). *P< 0.05 and **P< 0.01.

Previously, we have shown that miR-451 functions as a tumor suppressor in NSCLC by targeting ras-related protein 14 (RAB14). Here, the expression of RAB14 protein showed no difference between in docetaxel-resistant and parental LAD cells (data not shown). As miRNA can interact with hundreds of genes and a gene can be targeted by many miRNAs, miR-451 may play critical roles in radioresistance of docetaxel-resistant LAD cells by targeting other mRNAs. By performing a computational screen for genes with complementary sites of miR-451 in their 3'-UTR using open access softwares (TargetScan, miRBase targets and PicTarget), c-Myc might be a putative target of miR-451. The c-Myc protein, a nuclear phosphprotein with DNA binding properties, has a pivotal function in growth control, differentiation and apoptosis. First, qRT-PCR and Western blotting was performed to detect the expression of c-Myc mRNA and protein in docetaxel-resistant and parental LAD cells, and it was observed that c-Myc mRNA and protein was significantly upregulated in docetaxel-resistant LAD cells in comparison with the parental LAD cells (Figure 5A). To further determine whether c-Myc was a direct target of miR-451, we investigated the binding site of miR-451 in the 3'-UTR sequence of c-Myc mRNA (1891-1912 nt). We generated a luciferase reporter (pLUC/c-Myc/3'-UTR-wt) in which the nucleotides of the c-Myc-3'-UTR complementary to miR-451 were inserted into the pLUC vector, and we also generated a mutant reporter (pLUC/c-Myc/3'-UTR-mut). 48h after pcDNA/miR-451 (or pcDNA/miR-NC) and pLUC/c-Myc/3'-UTR-wt (or pLUC/c-Myc/3'-UTR-mut) were co-transfected into HEK293T cells, and then the luciferase activity was measured. The luciferase activity of the pLUC/c-Myc/3'-UTR-wt-transfected cells co-transfected with pcDNA/miR-451 was significantly decreased in comparison with that of the pLUC/c-Myc/3'-UTR-wt-transfected cells co-transfected with pcDNA/miR-NC (Figure 5B), suggesting that c-Myc might be a direct target of miR-451.

Recent studies have shown that rad-51 and survivin both of which are reported to be firmly linked with radioresistance of tumor cells can be transcriptionally activated by c-Myc [19, 20]. Rad-51, an important component of DNA homologous recombination system, plays key roles in repairing DNA DSBs which are the predominant lethal lesions induced by irradiation [21]. Survivin, a key regulator of apoptosis pathway, has been reported to be associated with cell survival and maintenance [22]. Here, qRT-PCR and Western blotting assays indicated that the mRNA and protein expression levels of rad-51 and survivin in SPC-A1/DTX and H1299/DTX cells were significantly higher than those in parental cells (Figure 5C). Then, we explored whether miR-451 was involved in the regulation of these radioresistance-related genes. As shown in Figure 5D, the expression levels of c-Myc, rad-51 and survivin both mRNA and protein were significantly decreased in pcDNA/miR-451-transfected SPC-A1/DTX or H1299/DTX cells in comparison with pcDNA/miR-NC-transfected cells. Also, the mRNA and protein expression levels of these genes were significantly upregulated in Anti-miR-451-transfected SPC-A1 or H1299 cells in comparison with Anti-miR-NC-transfected cells (Figure 5E).

**Figure 6:**
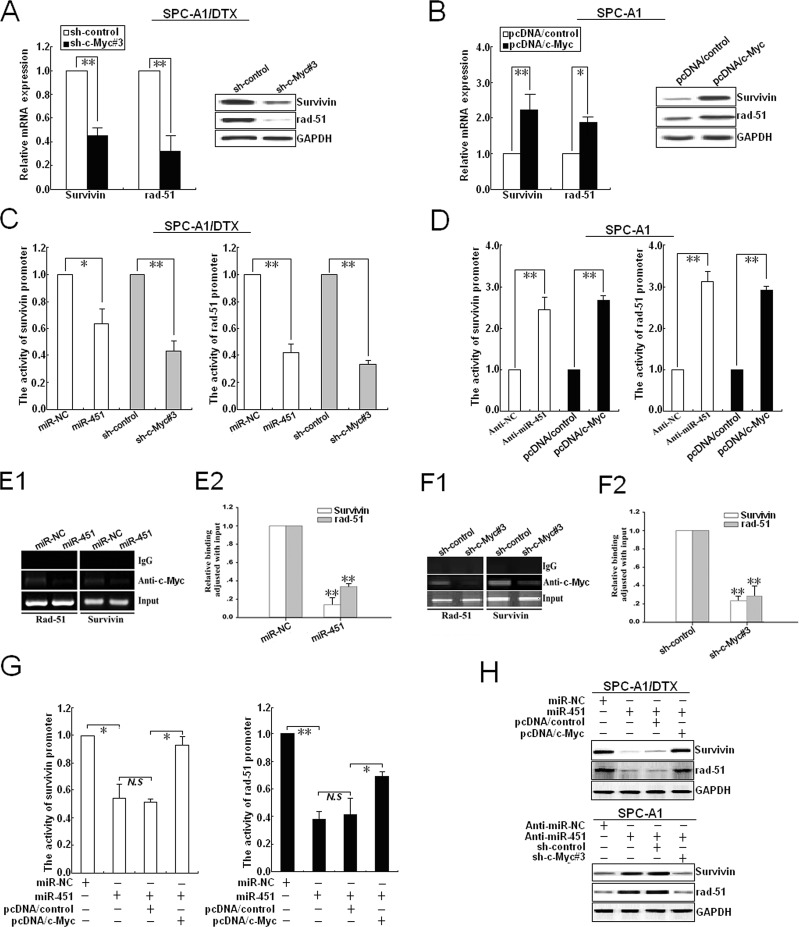
c-Myc transcriptionally activates rad-51 and survivin expression in LAD cells (A) qRT-PCR and Western blotting detection of rad-51 and survivin mRNA and protein expression in sh-c-Myc#3 (or sh-control) -transfected SPC-A1/DTX cells. (B) qRT-PCR and Western blotting detection of rad-51 and survivin mRNA and protein expression in pcDNA/c-Myc (or pcDNA/control)-transfected SPC-A1 cells. (C) Analysis of the reporter activity of survivin and rad-51 promoter (survivin or rad-51 promoter/Luc) in pcDNA/miR-451 (or pcDNA/miR-NC) and sh-c-Myc#3 (or sh-control)-transfected SPC-A1/DTX cells. Each cell type was transiently transfected with survivin or rad-51 promoter/Luc plasmid. Dual-luciferase reporter assays were performed on the lysed cells co-transfected with rad-51 or survivin promoter/Luc (firefly luciferase) and phRL-SV (hRenilla luciferase) 48 h after co-transfection. Reporter gene activation was determined as a relative ratio of firefly luciferase to hRenilla luciferase activity. (D) Analysis of the reporter activity of survivin and rad-51 promoter in An-miR-451 (or Anti-miR-NC) and pcDNA/c-Myc (or pcDNA/control)-transfected SPC-A1 cells used above-mentioned methods. (E1-2) Upregulation of miR-451 decreased the amount of c-Myc binding to the promoters of survivin and rad-51.ChIP assay was performed with antibody directly against c-Myc in SPC-A1/DTX cells transfected with pcDNA/control or pcDNA/miR-451, repectively. ChIP-derived DNA was amplified after immunoprecipitation by qRT-PCR with primers designed to amplify the sequences containing the putative c-Myc-binding sites. Data were adjusted with qRT-PCR products that were amplified with input DNA before immunoprecipitation and determined relative to miR-NC group. (F1-2) silencing of c-Myc decreased the amount of c-Myc binding to the promoters of survivin and rad-51. Chip assays were performed used above-mentioned methods. (G) Analysis of the reporter activity of survivin and rad-51 promoter in pcDNA/miR-NC or pcDNA/miR-451-transfected SPC-A1/DTX cells co-transfected with pcDNA/control or pcDNA/c-Myc used above-mentioned methods. (H) Western blotting detection of rad-51 and survivin protein expression in pcDNA/miR-NC or pcDNA/miR-451-transfected SPC-A1/DTX cells co-transfected with pcDNA/c-Myc (or pcDNA/control), or Anti-miR-451 (or Anti-miR-NC)-transfected SPC-A1 cells co-transfected with sh-control or sh-c-Myc#3. GAPDH was used as an internal control. Results represent the average of three independent experiments (mean±SD). *P< 0.05, **P< 0.01 and N.S >0.05.

To further confirm that c-Myc is involved in the regulation of rad-51 and survivin mediated by miR-451 in LAD cells, we first determine the effect of c-Myc expression on the expression of those proteins. First, sh-c-Myc (#1, #2 or #3) and sh-control vectors were stably transfected into SPC-A1/DTX or H1299/DTX cells, respectively, and results indicated that the inhibitory effect of sh-c-Myc#3 was the biggest (Supplementary Figure 3A-B). Silencing of c-Myc could lead to the decreased expression of survivin and rad-51 mRNA and protein in SPC-A1/DTX cells, while overexpression of c-Myc could increase their expression in parental SPC-A1 cells (Figure 6A-B). Then, the effects of miR-451/c-Myc on the activity of survivin and rad-51 promoters were further analyzed, and results showed that upregulation of miR-451 and silencing of c-Myc could lead to the reduced transcriptional activity of survivin and rad-51 promoters in SPC-A1/DTX cells (Figure 6C). Likewise, downregulation of miR-451 and upregulation of c-Myc could lead to the increased transcriptional activity of survivin and rad-51 promoters in SPC-A1 cells (Figure 6D). Furthermore, ChIP assays indicated that upregulation of miR-451 could reduce the amount of c-Myc binding to the promoters of survivin and rad-51 *in vivo* (Figure 6E1-2), while silencing of c-Myc could also reduce the amount of c-Myc binding to those promoters *in vivo* (Figure 6F1-2). Next, we determine the activity of survivin or rad-51 promoter reporters in pcDNA/miR-451-transfected SPC-A1/DTX cells co-transfected with pcDNA/c-Myc, and showed that overexpression of c-Myc could partially rescue the decreased activity of survivin or rad-51 promoter reporters induced by miR-451 upregulation (Figure 6G). Importantly, upregulation of c-Myc could partially rescue the decreased expression of survivin and rad-51 induced by miR-451 upregulation, while silencing of c-Myc could also partially rescue the increased expression of survivin and rad-51 induced by miR-451 downregulation (Figure 6H). Thus, c-Myc, identified as a direct target of miR-451, is responsible for regulation of rad-51 and survivin in docetaxel-resistant LAD cells.

### Silencing of c-Myc phenocopies the effect of miR-451 on reversing the radioresistance of docetaxel-resistant LAD cells both *in vitro* and *in vivo*

**Figure 7:**
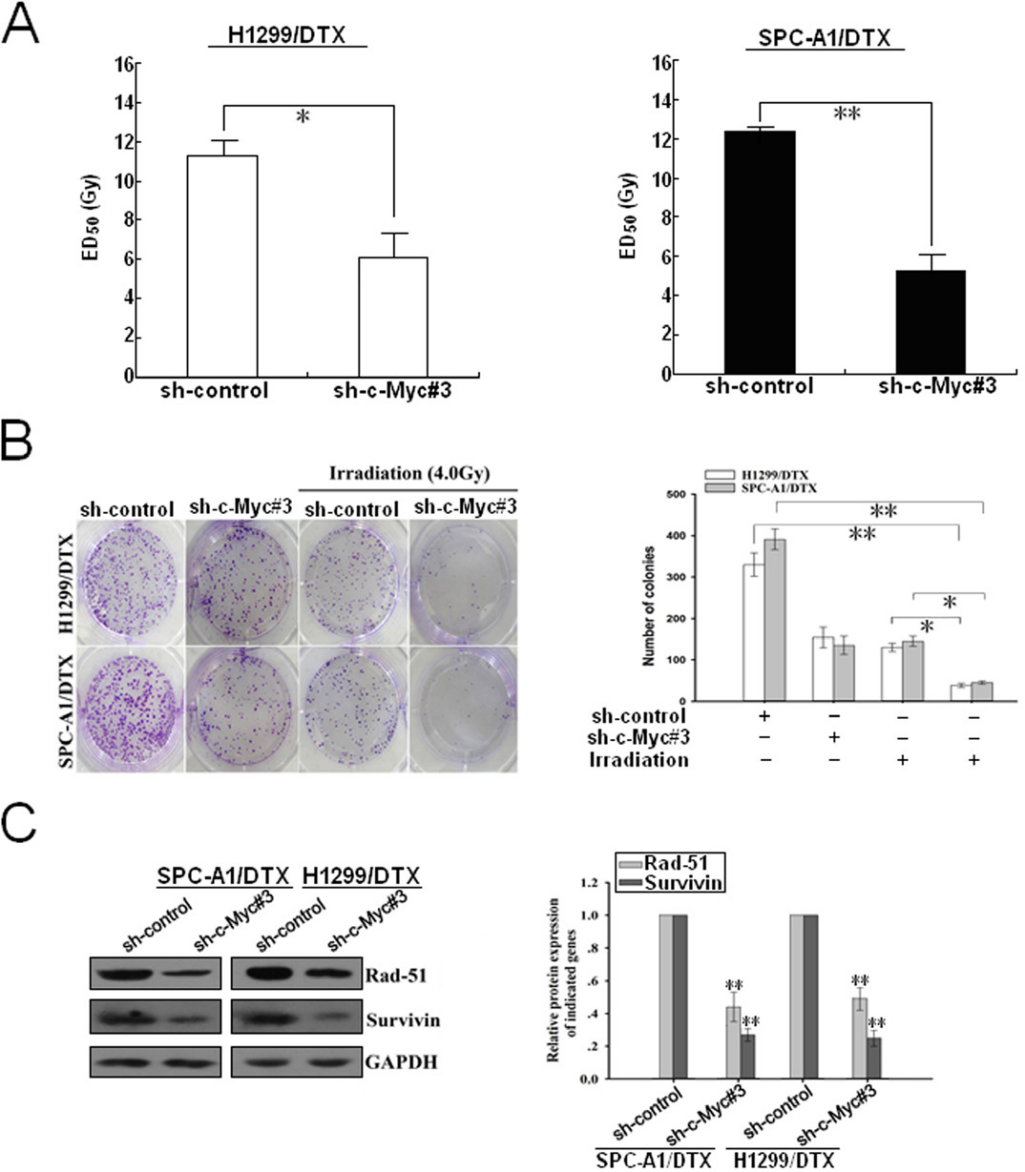
Silencing of c-Myc increases the sensitivity of docetaxel-resistant LAD cells to irradiation (A) CCK-8 assay was conducted to detect the ED_50_ value of irradiation to sh-control or sh-c-Myc#3-transfected H1299/DTX or SPC-A1/DTX cells. (B) The colony formation of sh-c-Myc#3 (or sh-control)-transfected H1299/DTX or SPC-A1/DTX cells treated without irradiation or with irradiation (4.0 Gy). (C) Western blotting detection of rad-51 and survivin protein expression in sh-c-Myc#3 (or sh-control)-transfected H1299/DTX or SPC-A1/DTX cells. GAPDH was used as an internal control. Results represent the average of three independent experiments (mean±SD). *P< 0.05 and **P< 0.01.

As the pro-oncogene c-Myc was identified as a functional target of miR-451, we then focused on understanding the effect of c-Myc expression on radioresistance of docetaxel-resistant LAD cells. First, the ED_50_ values of irradiation in docetaxel-resistant LAD cells stably transfected with sh-control or sh-c-Myc#3 vector were determined by CCK-8 assay. As shown in Figure 7A, the ED_50_ values of irradiation in shRNA-c-Myc#3-transfected SPC-A1/DTX or H1299/DTX cells were significantly reduced, compared with those in sh-control-transfected cells (*P*<0.01). Also, silencing of c-Myc could significantly reduce the colony formation capacity of SPC-A1/DTX and H1299/DTX cells in the presence of irradiation (Figure 7B). Furthermore, silencing of c-Myc could also lead to the decreased expression of rad-51 and survivin proteins in SPC-A1/DTX and H1299/DTX cells (Figure 7C). Next, we explored whether silencing of c-Myc could recapitulate the effect of miR-451 upregulation on irradiation-induced apoptosis in docetaxel-resistant LAD cells. When combined with irradiation treatment (4.0Gy), the apoptotic rate of sh-c-Myc#3-transfected SPC-A1/DTX or H1299/DTX cells was significantly increased compared with those of sh-control-transfected cells (Supplementary Figure 4A). Then, Western blotting was performed to detect the expression of cleaved or total caspase-3 proteins. When combined with irradiation treatment (4.0Gy), the expression level of cleaved caspase-3 protein in sh-c-Myc#3-transfected SPC-A1/DTX or H1299/DTX cells was significantly increased in comparison with sh-control-transfected cells, but the expression of total caspase-3 protein showed no difference (Supplementary Figure 4B). In addition, silencing of c-Myc combined with irradiation treatment (4.0Gy) significantly increased γ-H2A.X foci formation compared with control group (Supplementary Figure 5A). The expression of γ-H2A.X protein was upregulated in sh-c-Myc#3-transfected cells compared with that in control group, when combined with irradiation treatment (4.0Gy) (Supplementary Figure 5B). Collectively, silencing of c-Myc mimics the effects of miR-451 upregulation on enhancing irradiation-induced apoptosis and DNA DSBs in docetaxel-resistant LAD cells.

### Overexpression of c-Myc rescues the effects of enhanced miR-451 expression on on radioresistance of docetaxel-resistant LAD cells

**Figure 8:**
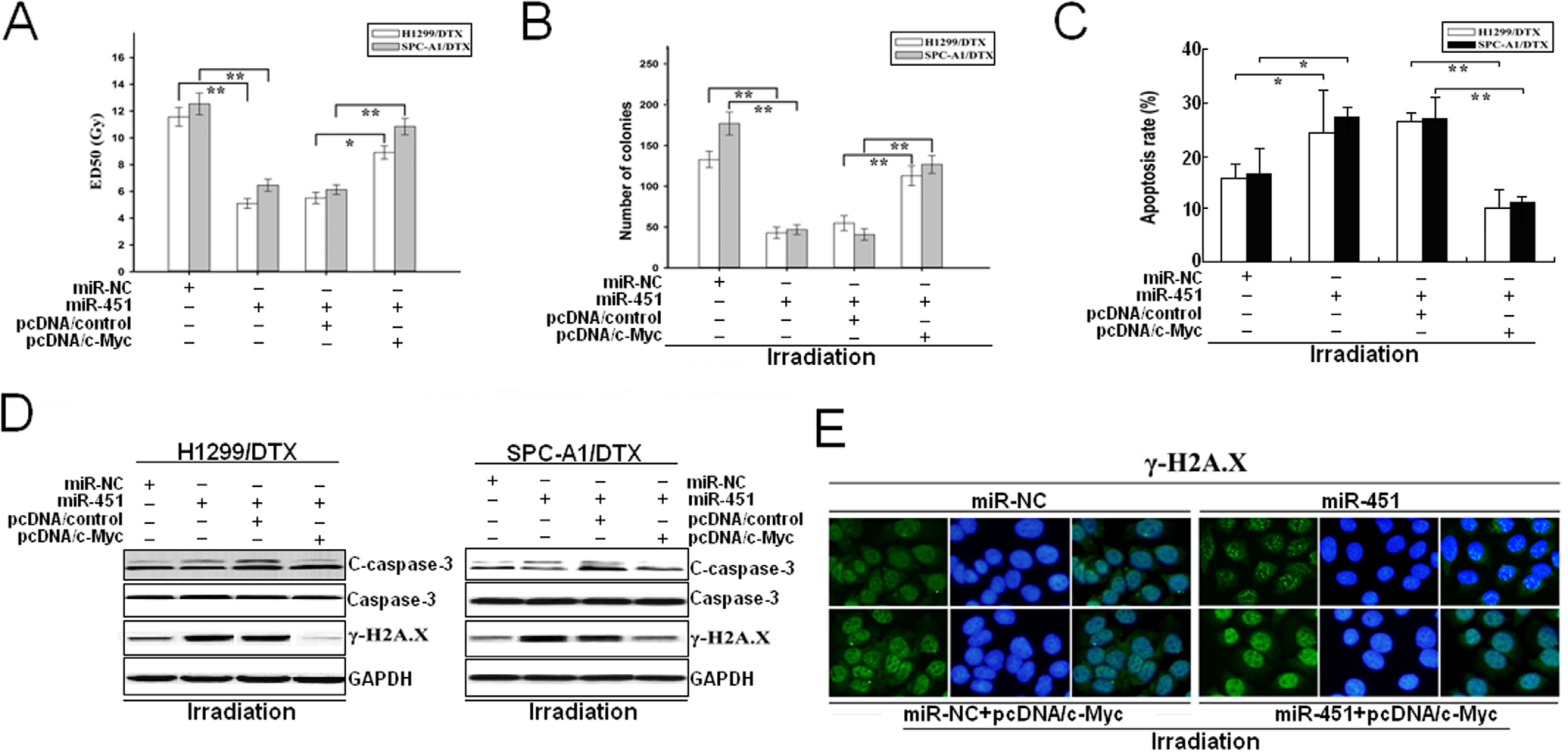
Overexpression of c-Myc partially reverses the effects of miR-451 upregulation on radioresistance of docetaxel-resistant LAD cells (A) CCK-8 assay was conducted to detect the ED_50_ values of irradiation in pcDNA/miR-451 (or pcDNA/miR-NC)-transfected H1299/DTX or SPC-A1/DTX cells co-transfected with pcDNA/control or pcDNA/c-Myc. (B) The colony formation of pcDNA/miR-451 (or pcDNA/miR-NC)-transfected H1299/DTX or SPC-A1/DTX cells combined with pcDNA/control or pcDNA/c-Myc co-transfection and irradiation treatment (4.0Gy). (C) Flow cytometry detection of apoptosis in pcDNA/miR-451 (or pcDNA/miR-NC)-transfected H1299/DTX or SPC-A1/DTX cells combined with pcDNA/control or pcDNA/c-Myc co-transfection and irradiation treatment (4.0Gy). (D) Western blotting detection of C-caspase-3, total Caspase-3 and γ-H2A.X protein expression in pcDNA/miR-451 (or pcDNA/miR-NC)-transfected H1299/DTX or SPC-A1/DTX cells combined with pcDNA/control or pcDNA/c-Myc co-transfection and irradiation treatment (4.0Gy). GAPDH was used as an internal control. (E) Immunofluorescence detection of phosphorylation of γ-H2A.X foci formation in pcDNA/miR-451(or pcDNA/miR-NC)-transfected SPC-A1/DTX cells combined with pcDNA/control or pcDNA/c-Myc co-transfection and irradiation treatment (4.0Gy). Results represent the average of three independent experiments (mean±SD). *P< 0.05 and **P< 0.01.

To further confirm that c-Myc was involved in miR-451-mediated chemo- and radiotherapy cross resistance in LAD cells, pcDNA/c-Myc or pcDNA/control was transfected into docetaxel-resistant LAD cells stably transfected with pcDNA/miR-451 or pcDNA/miR-NC. 48 hours after transfection, it was observed that pcDNA/c-Myc could partially rescue the decreased ED_50_ values of irradiation in SPC-A1/DTX or H1299/DTX cells induced by miR-451 upregulation (Figure 8A). Also, upregulation of c-Myc could partially abrogate the decreased colony-formation capacity of pcDNA/miR-451-transfected SPC-A1/DTX or H1299/DTX cells with irradiation treatment (4.0Gy) (Figure 8B). Furthermore, we determine whether c-Myc overexpression could rescue miR451-induced promotion of apoptosis and DNA DSBs. Flow cytometry assay showed that c-Myc overexpression could rescue the increased apoptosis of pcDNA/miR-451-transfected SPC-A1/DTX or H1299/DTX cells combined with irradiation treatment (4.0Gy) (Figure 8C). Also, Western blotting assay further indicated that overexpression of c-Myc could rescue the increased expression of cleaved caspase-3 and γ-H2A.X proteins in pcDNA/miR-451-transfected SPC-A1/DTX or H1299/DTX cells combined with irradiation treatment (4.0Gy) (Figure 8D). Additionally, immunofluorescence analysis of γ-H2A.X foci formation also confirmed the results of Western blotting assay (Figure 11E). Therefore, overexpression of c-Myc partially rescues the effects of miR-451 upregulation on radioresistance of docetaxel-resistant LAD cells.

### c-Myc is inversely correlated with miR-451 and positively correlated with rad-51 and survivin in LAD tissues

qRT-PCR was performed to detect the mRNA expression of miR-451, rad-51 and survivin in 32 paired of LAD and corresponding nontumor tissues. In consensus with our previous report [26], miR-451 was found to be predominantly downregulated in LAD tissues, compared with corresponding nontumor tissues (Supplementary Figure 6A). Meanwhile, the expression mRNA levels of c-Myc, rad-51 and survivin were significantly upregulated in LAD tissues, compared with corresponding nontumor tissues (Supplementary Figure 6B-D). In the same LAD tissues, we then correlated miR-451 with the mRNA expression levels of c-Myc, rad-51 and survivin. Significant inverse correlations were observed when three mRNA expression levels (c-Myc, rad-51 and survivin) were plotted against miR-451 expression levels (2-tailed Spearman's correlation; miR-451/c-Myc: r = −0.754, *P*<0.0001; miR-451/rad-51: r = −0.599, *P*<0.0001; miR-451/survivin: r = −0.662, *P*<0.0001) (Supplementary Figure 4E-G). In the same LAD tissues, we then correlated c-Myc with the mRNA expression levels of rad-51 and survivin. Significant positive correlations were observed when two mRNA expression levels (rad-51 and surviving) were plotted against c-Myc mRNA expression levels (2-tailed Spearman's correlation; c-Myc/rad-51: r=0.779, *P*<0.001; c-Myc/survivin: r=0.889, *P*<0.001) (Supplementary Figure 4H-I). These data further support the existence of a novel miR-451/c-My-rad-51/survivin signaling pathway in LAD.

## DISCUSSION

In the present study, for the first time, we showed that downregulation of miR-451 was significantly correlated with chemo- and radiotherapy cross resistance in LAD cells. Re-expression of miR-451 could reverse radioresistance of docetaxel-resistant LAD cells through promoting apoptosis and DNA DSBs. The proto-oncogene c-Myc was identified as a direct target of miR-451, and re-expression of miR-451 significantly inhibited the expression of survivin and rad-51 by reducing the amount of c-Myc protein binding to the promoters of survivin and rad-51. To the best of our knowledge, this is the first report about the involvement of miR-451/c-Myc-survivin/rad-51 signaling in chemo- and radiotherapy cross resistance of LAD cells.

Chemoresistant tumors often fail to respond to other cytotoxic treatments such as radiotherapy [22]. However, the molecular mechanisms of chemo- and radiotherapy cross resistance are not fully understood. Recent evidence has shown that non-coding RNAs (ncRNAs) play an important role in tumor pathogenesis, and provide new insights into the biology of chemo- and radiotherapy cross resistance [23]. Over the past decade, miRNAs have moved to the forefront of ncRNA research in human cancers. MiRNAs, a class of small endogenous noncoding RNAs, make up a novel class of gene regulators [24]. Increasing evidence has firmly shown that miRNAs regulate a variety of cellular processes such as differentiation, development, proliferation and metabolism [25-28]. Recent evidence indicates that the aberrant regulation of miRNAs plays an important role in pathogenesis of lung cancer, including chemo- or radioresistance [29, 30]. In previous study, we have analyzed the miRNA expression profiles in NSCLC by use of a miRNA microarray platform and identified 40 differentially expressed miRNAs, and miR-451 was found to be the most downregulated. Also, miR-451 was found to be significantly correlated with tumor differentiation, pathological stage, lymph node metastasis and poor prognosis of patient, and further researches indicated that miR-451 could inhibit growth and enhance apoptosis of NSCLC cells by targeting RAB14 [15]. At the same time, the tumor suppressor functions of miR-451 in other types of human malignancies are increasingly reported. Godlewski' et al identified a potential feedback loop between LKB1 and miR-451, and showed that microRNA-451 is a conditional switch controlling glioma cell proliferation and migration [31]. Li and his colleagues showed that miR-451 inhibits growth of human colorectal carcinoma cells via downregulation of PI3k/Akt pathway [32]. In another report, the over-expressed miR-451 in colon cancer cells was found to inhibit AMPK to activate mTORC1, which mediates FSCN1 expression and cancer cell progression [33]. Meanwhile, miR-451 is reported to be involved in the self-renewal, tumorigenicity, and chemoresistance of colorectal cancer stem cells [34]. The associations of miR-451 with chemo- or radiosensitivity of tumor cells are also reported. Upregulation of miR-451 not only increases cisplatin sensitivity of non-small cell lung cancer cell line, but also reverses the resistance of the MCF-7 breast cancer cells to chemotherapeutic drug doxorubicin [35, 36]. Interestingly, overexpression of miR-451 in gastric and colorectal cancer cells reduced cell proliferation and increased sensitivity to radiotherapy by regulation of macrophage migration inhibitory factor production [37]. However, whether miR-451 plays a critical role in chemo- or radiotherapy cross resistance in LAD cells is still unclear.

To further investigate the molecular mechanisms of docetaxel resistance and provide therotical support for drug resistant reversal induced by docetaxel, the multidrug-resistant LAD cell lines (SPC-A1/DTX and H1299/DTX) were previously established from human LAD cell lines (SPC-A1 and H1299) in our lab by stepwise selection using docetaxel as inducing reagent. It was observed that docetaxel-resistant LAD cells not only showed the increased chemoresistance but also was cross-resistant to irradiation. Thus, establishment of docetaxel-resistant LAD cell models will provide foundation for further research on the molecular mechanisms involved in the formation of radioresistance in chemoresistant LAD cells. Here, miR-451 was found to be significantly lower in docetaxel-resistant LAD cells, compared with parental LAD cells. By gain- and loss-of-function assays, upregulation of miR-451 significantly increases the sensitivity of docetaxel-resistant LAD cells to irradiation both *in vitro* and *in vivo* by enhancing DNA DSBs and apoptosis. DNA DSBs have been regarded as the major lethal lesions involved in irradiation-mediated cell deaths [38]. Thus, the successful repair of DNA DSBs induced by irradiation is critical for preserving genomic integrity and maintenance of cell survival, and the defective DNA DSBs repair of cancer cells can be exploited for cancer therapy combined with irradiation [39]. However, irradiation-mediated DNA DSBs can be efficiently repaired by DNA homologous recombination which needs numerous factors including the central recombinase rad-51 [40]. Recent studies have highlighted that silenced rad-51 is correlated with increased radiosensitivity of tumor cells and targeting rad-51 will be a potential target for combined therapies in clinic [41, 42]. Previously, aberrant expression of miRNAs has been reported to be associated with tumor radioresistance. For example, overexpressed miR-155 could reduce the efficiency of DNA repair and enhance radiosensitivity of breast cancer cells by directly repressing RAD51 [43]. Upregulation of miR-449a effectively increases irradiation-induced DNA damage and apoptosis, and eventually enhances radiosensitivity of CL1-0 LAD cells [44]. Here, we showed that upregulation of miR-451 significantly decreased protein expression of rad-51, which eventually led to the increased DNA DSBs (formation of γ-H2A.X foci) in docetaxel-resistant LAD cells. It is well established that inhibition of cell growth by promoting apoptosis is one of the mechanisms responsible for radiotherapy [45]. Survivin, a key regulator of apoptosis pathway has been linked to increased tumor cell survival potential by inhibiting apoptosis, and knockdown of survivin enhances radiosensitivity of breast cancer cells [46]. In this study, our data indicated that enforced miR-451 expression significantly reduced survivin expression, which led to the increased caspase-3-dependent apoptosis. Therefore, re-expression of miR-451 enhances DNA DSBs and apoptosis by downregulating rad-51 and survivin, which finally leads to the reversal of radioresistance in docetaxel-resistant LAD cells.

To further understand the molecular mechanisms involved in regulation of rad-51 and survivin of docetaxel-reistant LAD cells induced by miR-451, the determination of its direct and functional target genes is needed. RAB14 belongs to the large RAB family of low molecular mass GTPases that are involved in intracellular membrane trafficking [47]. In our previous study, RAB14 has been identified as a functional target of miR-451 in NSCLC, but the expression of RAB14 showed no difference between docetaxel-resistant and parental LAD cells (data not shown). As one microRNA can regulate much target mRNAs and one mRNA can also be regulated by much microRNAs. Thus, miR-451 might regulate chemo- or radiotherapy cross resistance of LAD cells by targeting other mRNAs. By using three algorithms, c-Myc was predicated to a potential target of miR-451, which was consistent with the previous study [48]. The proto-oncogene c-Myc, a critical regulator of tumor cell growth, apoptosis and DNA DSBs repair has been associated with radioresistance of numerous tumors [49]. Overexpressed c-Myc leads to activation of the DNA-damage-checkpoint response and increased radioresistance of the PKH26+ stem cell-like subpopulation from nasopharyngeal carcinoma, and knockdown of c-Myc significantly increases DNA DSBs induced by irradiation in the PKH26+ stem cell-like cells [50]. In this study, we showed that upregulation of miR-451 significantly reduced the luciferase activity of the c-Myc-3'-UTR reporter and decreased protein expression of c-Myc, suggesting that c-Myc might be a conserved target gene of miR-451. Recently, c-Myc has been reported to transcriptionally activate rad-51 and survivin both of which are firmly linked to radiosensitivity of tumor cells. Here, both re-expression of miR-451 and silencing of c-Myc could significantly downregulate the expression of rad-51 and survivin by reducing the amount of c-Myc binding to the promoters of survivin and rad-51. Furthermore, silencing of c-Myc could phenocopy the effect of miR-451 upregulation on the reversal of radioresistance in docetaxel-resistant LAD cells by promoting apoptosis and DNA DSBs. More importantly, overexpression of c-Myc could partially rescue the effects of enhanced miR-451 on docetaxel-resistant LAD cells. Our data are supported by clinical data where we found a statistically significant inverse correlation between the expression of miR-451 and c-Myc, rad-51 or survivin in LAD tissue samples. Therefore, dysregulation of miR-451/c-Myc-rad-51/survivin signaling might be correlated with chemo- and radiotherapy cross resistance of LAD cells.

**Figure 9:**
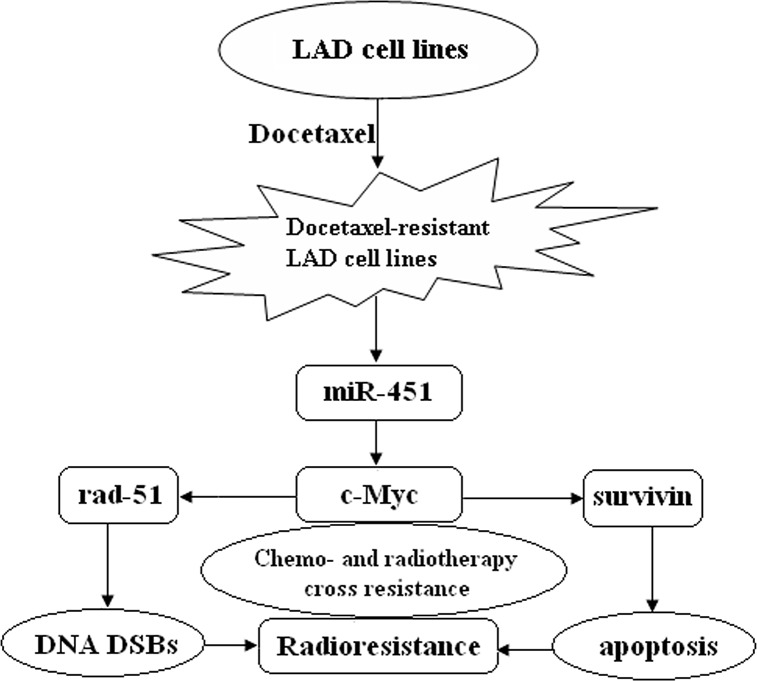
A proposed model for miR-451/c-Myc-rad-51/survivin signaling pathway in chemo- and radiotherapy cross resistance in LAD cells Docetaxel-resistant LAD cells acquired radioresistant phenotype where miR-451 was downregulated, resulting in chemo- and radiotherapy cross resistance.

In summary, our study showed mechanistically for the first time how cellular miRNA regulates chemo- and radiotherapy cross resistance in LAD cells. Present data demonstrate that re-expression of miR-451 downregulates the expression of rad-51 and survivin by targeting c-Myc, which promotes DNA DSBs and induces apoptosis enhancement, and eventually reverses radioresistance of docetaxel-resistant LAD cells. Therefore, loss of miR-451 during chemo- and radiotherapy cross resistance of LAD cells is likely to lead mis-regulation of this network (Figure 9). Thus, this newly identified miR-451/c-Myc-rad-51/survivin signaling pathway provides novel insight into the molecular mechanisms which regulate chemo- and radiotherapy cross resistance, and further provides a promising strategy for the treatment of chemoresistant LAD in future.

## MARTERIALS AND METHODS

### Cell culture

The parental human LAD cell lines (SPC-A1 and H1299) were obtained from the Cell Bank of Shanghai institute of cell biology (Chinese Academy of Medical Sciences,Shanghai, China). The docetaxel-resistant LAD cells (SPC-A1/DTX and H1299/DTX) derived from parental SPC-A1 and H1299 cells, respectively, were established and cultured as described in our previous work [16, 17].

### Cell transfection

pcDNA/miR-451 (or pcDNA/miR-NC) and Anti-miR-451 (or Anti-miR-NC) were used as our previously described [10]. Short hairpin RNA (shRNA) specifically targeting human c-Myc (GenBank no. NM_002467) was designed to knockdown c-Myc expression. The target sequence of shRNA targeting c-Myc and a negative control shRNA were listed in Supplementary Table 2. All the above sequences were inserted into the BglII and HindIII enzyme sites of pSilencer4.1-CMVneo vector, respectively. The recombinant plasmids were named sh-control, sh-c-Myc#1, sh-c-Myc#2 and sh-c-Myc#3, respectively. All of the vectors were confirmed by DNA sequencing. The cell transfection was performed in opti-MEM with the transfection reagent Lipofectamine™ 2000 (Invitrogen, CA, USA) following the manufacturer's instructions. For stable transfection, the cell lines transfected with pcDNA/miR-451 (or pcDNA/miR-NC) or sh-c-Myc#1, 2 or 3 (sh-control) vector were stably selected with G418 (400 mg/mL). 48 h later after transfection, and individual clones were isolated and maintained in a medium containing G418 (100 mg/mL).

### 3-(4,5-Dimethyl-2-thiazolyl)-2,5-diphenyl-2H-tetrazolium bromide (MTT) assay

The cells were seeded into 96-well culture plates. After overnight incubation, cells were treated with various concentrations of chemotherapeutic drugs. Following incubation for 24h, cell growth was measured following addition of 0.5 mg/ml MTT solution (Sigma). About 4 h later, the medium was replaced with 100μL dimethylsulfoxide (DMSO, Sigma) and vortexed for 10 min on horizontal oscillator. Absorbance was then recorded at 490 nm using a microplate reader (Bio-Rad, USA).

### *In vitro* radiosensitivity assay

Radiosensitivity assay was measured with Cell Counting Kit-8 (CCK-8) assay (Dojindo, Japan) according to the manufacturer's instructions. Briefly, 3.0×10^3^ cells were seeded into 96-well plates directly or 24 hours after transfection. After attachment, cells were treated with various doses of irradiation. 48 hours later, 10 μL of CCK-8 was added and incubated for 4 hours at 37°C. Absorbance was then measured at 450 nm with a microplate reader (Bio-Rad, USA). All experiment were performed in triplicate and repeated at least three times.

### Colony formation assay

A total of 1000 cells were seeded into 6-well plates directly or 24 hours after transfection. Two weeks later, cells were fixed with pure methanol and stained with 0.1% crystal violet. Visible colonies were then manually counted.

### Real-time quantitative reverse-transcription polymerase chain reaction (qRT-PCR) assay

Total cellular RNA was extracted with TRIzol reagent (Takara, Japan). Reverse transcription was performed with PrimeScript RT reagent Kit (Takara, Japan) according to the manufacturer's instructions. qRT-PCR was performed with SYBR Prime Script RT-PCR Kits (Takara, Japan) according to the manufacturer's instructions. The miR-451 or mRNAs level was calculated with the 2^−ΔΔCt^ method using U6 rRNA, respectively, as the reference genes. The expression levels were measured relative to the fold change of the control cells that was defined as 1.0. All experiments were conducted in triplicate. The primer pairs were presented in Supplementary Table 1.

### Flow cytometric analysis of apoptosis

Flow cytometric analysis of apoptosis was performed with Annexin V: FITC Apoptosis Detection Kits (BD Biosciences, USA) respectively, according to the manufacturer's instructions.

### Western blotting assay

Total protein lysate was separated by 10% sodium dodecyl sulfate-polyacrylamide gel electrophoresis (SDS-PAGE). And, proteins were transferred onto polyvinylidene fluoride (PVDF) membranes (Millipore, USA). Next, the membranes were blocked with 10% skim milk in TBST for 2 hours at room temperature and probed with specific primary antibodies overnight at 4°C. Then, the membranes were washed by TBST [120 mM Tris-HCl (pH 7.4), 150 mM NaCl, and 0.05% Tween 20], and incubated with secondary antibodies at room temperature for 2 hours. Next, the membranes were washed by TBST, and visualized with a chemiluminescence kit (Thermo Scientific, USA). The primary antibodies against Cleaved caspase-3 (C-caspase-3) (1:500 dilution), total Caspase-3 (1:200 dilution), γ-H2A.X (1:150 dilution), rad-51 (1:100 dilution), and surviving (1:200 dilution) (Abcam, HongKong), c-Myc (1:100 dilution) and GAPDH (1:200 dilution) (Santa Cruz Biotechnology, USA) were used in this study. GAPDH was used as an internal control.

### Immunofluorescence assay

Cells were placed on sterilized cover slips directly or 24 hours after transfection. After attachment, cells were treated with irradiation (4.0 Gy). 4 hours later, the cells were fixed in ice-cold acetone for 15 minutes, washed with PBS, and then stained with rabbit anti-γ-H2A.X (Abcam) overnight at 4°C after blocking with 3% BSA (Bovine serum albumin) for 30 minutes at room temperature. Then after washing, the cells were applied with goat anti-rabbit FITC conjugated secondary antibody for 30 minutes at room temperature and then counterstained with 4-6-diamidino-2-phenylindole for 2 minute at room temperature. The Images were captured with a fluorescent microscope.

### *In vivo* radiotherapy assay and immunostaining

BALB/c athymic nude mice (Male, SPF, 5-6 weeks) were provided by the department of comparative medicine of Jinling hospital. The in-vivo study was ethically approved and performed according to the institutional guidelines. Approximately 3.0×10^6^ SPC-A1/DTX/miR-NC or SPC-A1/DTX/Let-7c cells were suspended in 100 μL PBS and injected subcutaneously into the right side of the posterior flank of female BALB/c athymic nude mice (Department of comparative medicine, Jinling Hospital, Nanjing, China) at 5 to 6 weeks of age. Tumor growth was examined every other day with a vernier caliper. Tumor volumes were calculated by using the equation: V= A×B^2^/2 (mm^3^), wherein A is the largest diameter, and B is the perpendicular diameter. When the average tumor size reached about 50 mm^3^, the tumor-bearing nude mice were exposed to X-ray of 2.0 Gy alone for each time. The same treatment for each group were repeated 3 times (the interval time was 5 days). After 6 weeks, all mice were killed, and necropsies were performed. The primary tumors were excised, paraffin-embedded, formalin-fixed, and performed H&E staining, immunostaining analysis for PCNA and Ki-67 (proliferating cell nuclear antigen) protein expression and TUNEL staining detection of apoptosis according to the manufacturer's instructions.

### Luciferase reporter assay

Luciferase reporter containing wild type 3'-UTR of c-Myc (pLUC/c-Myc/3'-UTR-wt) in which the nucleotides of the c-Myc-3'-UTR complementary to miR-451 were inserted into the pGL3-Basic vector, and we also generated a mutant reporter (pluc/c-Myc/3'-UTR-mut) from the wild type with point mutation. The primers were presented in Supplementary Table 2. A total of 2.0×10^3^/well SPC-A1/DTX cells were seeded into 96-well plates and then co-transfected with the specific luciferase reporter plasmids, pcDNA/miR-NC or pcDNA/miR-451. Renila-TK plasmid (Promega, USA) was co-transfected into all samples. 48 hours after transfection, luciferase activities were measured with Dual-Luciferase Reporter Assay kits (Promega, USA). The luciferase activities were normalized by renilla luciferase activities. The data were relative to the fold change of the corresponding control groups that was defined as 1.0. All experiments were performed in triplicate.

### Chromatin immunoprecipitation (ChIP) assay

ChIP assay was performed with Immunoprecipitation Assay Kits (Millipore, USA) according to the manufacturer's instructions. Briefly, cells were cross-linked with 1% formaldehyde for 10 min at 37°C. The cells were then resuspended in 200 μl of lysis buffer and incubated for 10 minutes on ice. The lysate was sheared to lengths between 200 and 1000 base pairs by sonication. The supernatant was pre-cleared with a Salmon Sperm DNA/Protein A Agarose-50% Slurry. The recovered supernatant was incubated with anti-c-Myc antibody (Abcam) or an isotype control IgG overnight at 4ºC with rotation. Then, the antibody/DNA complex was collected using Salmon Sperm DNA/Protein A Agarose Slurry for one hour at 4ºC with rotation. The complex was eluted by elution buffer. Then, the crosslinks were reversed with 5M NaCl heating at 65ºC for 4 hours. The DNA sample was then purified and measured by qRT-PCR. The primers were listed on Supplementary Table 3.

### Patients and tissue samples

A total of 32 patients who were diagnosed as primary LAD in the Department of Cardiothoracic Surgery of Nanjing Military Medical Hospital from 2010 to 2012 were included in this study. None of these patients received chemotherapy and radiotherapy before the surgery. The detailed clinicopathological factors of patients were shown in Supplementary Table 4. Tumor and corresponding nontumor lung tissue samples were collected. Except tissues used for RNA extraction, the remnant tissues were rapidly frozen in liquid nitrogen and stored at −80°C. Ethical approval was obtained from the hospital and fully informed consent from all patients prior to sample collection.

### Statistical analysis

The data were the means ± standard error of at least three independent experiments. The SPSS 17.0 software (SPSS Inc., Chicago, IL, USA) was applied for statistical analysis. Multiple group comparisons were analyzed with one-way ANOVA and two group comparisons were performed with a Student *t* test. All tests performed were two-sided. *P* < 0.05 was considered statistically significant.

### SUPPLEMENTARY FIGURES AND TABLES


