# Supplementary material for: Acquisition of radioresistance in docetaxel-resistant human lung adenocarcinoma cells is linked with dysregulation of miR-451/c-Myc-survivin/rad-51 signaling
Source: Oncotarget. 2014 Jul 7;5(15):6113–29. doi: 10.18632/oncotarget.2176 (PMC4171617; doi:10.18632/oncotarget.2176)
Supplement: Supplementary file 1 [file oncotarget-05-6113-s001.pdf]

Acquisition of radioresistance in docetaxel-resistant human lung adenocarcinoma cells is linked with dysregulation of miR-451/c-Myc-survivin/rad-51 signaling

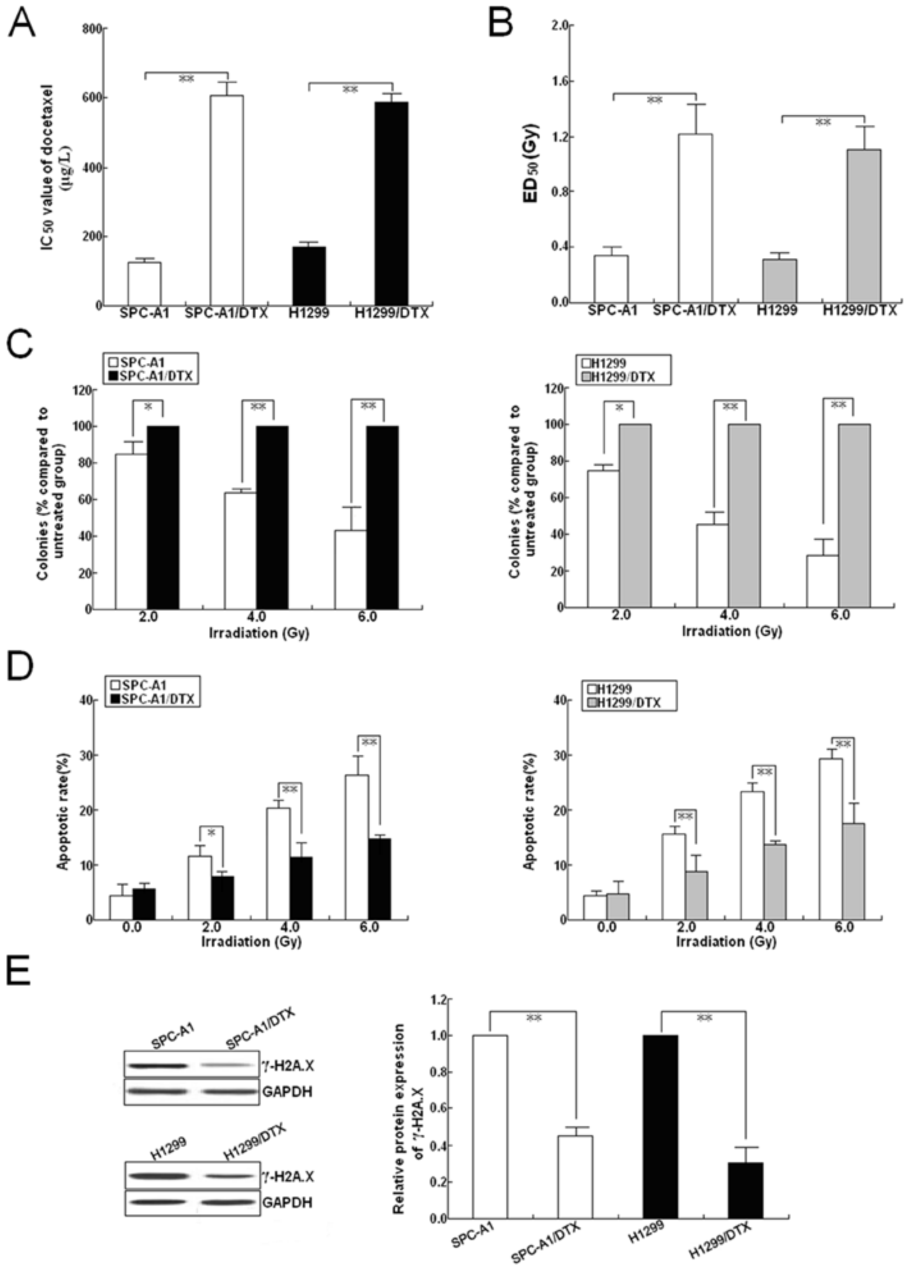

Supplementary Figure 1: Docetaxel-resistant LAD cells is cross-resistant to irradiation. (A) MTT assay was conducted to detect the IC_{50} values of docetaxel to docetaxel-resistant LAD cells (SPC-A1/DTX and H1299/DTX) and their parental LAD cells (SPC-A1 and H1299), respectively. (B) CCK-8 assay was conducted to detect the ED_{50} values of irradiation to docetaxel-resistant LAD cells (SPC-A1/DTX and H1299/DTX) and their parental LAD cells

(SPC-A1 and H1299), respectively. (C) The colony formation of docetaxel-resistant and parental LAD cells treated with various doses of irradiation (2.0, 4.0, and 6.0 Gy). (D) Flow cytometric analysis of apoptosis in docetaxel-resistant and parental LAD cells in the presence of the indicated doses of irradiation (0.0, 2.0, 4.0 and 6.0 Gy). (E) Western blotting detection of γ-H2A.X protein in docetaxel-resistant and parental LAD cells. Results represent the average of three independent experiments (mean±SD). $^{*}P < 0.05$ and $^{**}P < 0.01$.

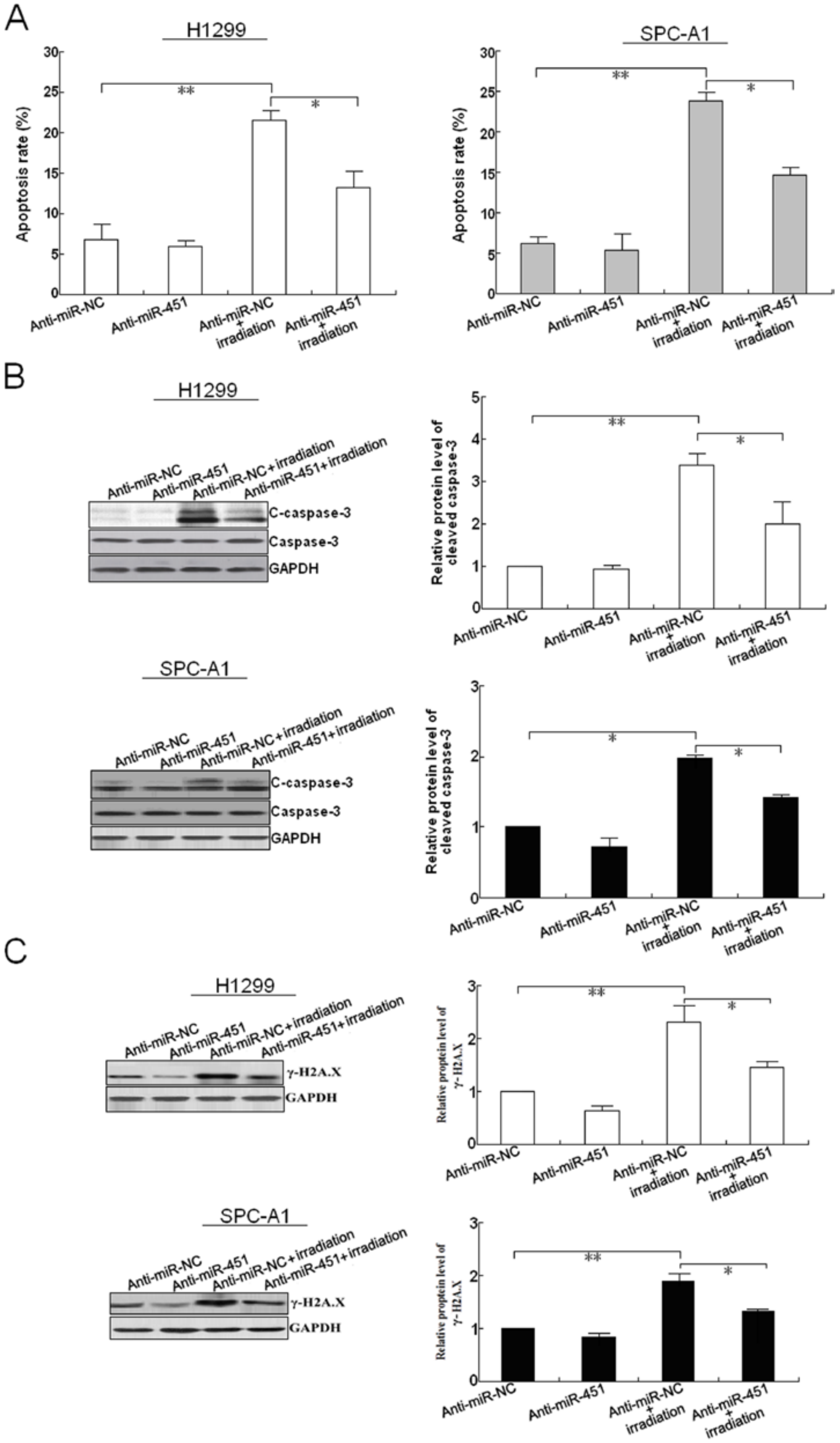

Supplementary Figure 2: Effect of Anti-miR-451 on radiosensitivity of parental LAD cells. (A) Flow cytometry detection of apoptosis in Anti-miR-451 (or Anti-miR-NC)-transfected H1299 or SPC-A1 cells combined with irradiation treatment (2.0Gy). (B) Western blotting

detection of C-caspase-3 and Caspase-3 protein expression in Anti-miR-451 (or Anti-miR-NC)-transfected H1299 or SPC-A1 cells combined with irradiation treatment (2.0Gy). GAPDH was used as an internal control. (C) Western blotting detection of γ-H2A.X protein expression in Anti-miR-451 (or Anti-miR-NC)-transfected H1299 or SPC-A1 cells combined with irradiation treatment (2.0Gy). GAPDH was used as an internal control. Results represent the average of three independent experiments (mean ± SD). $^{*}P< 0.05$ and $^{**}P< 0.01$.

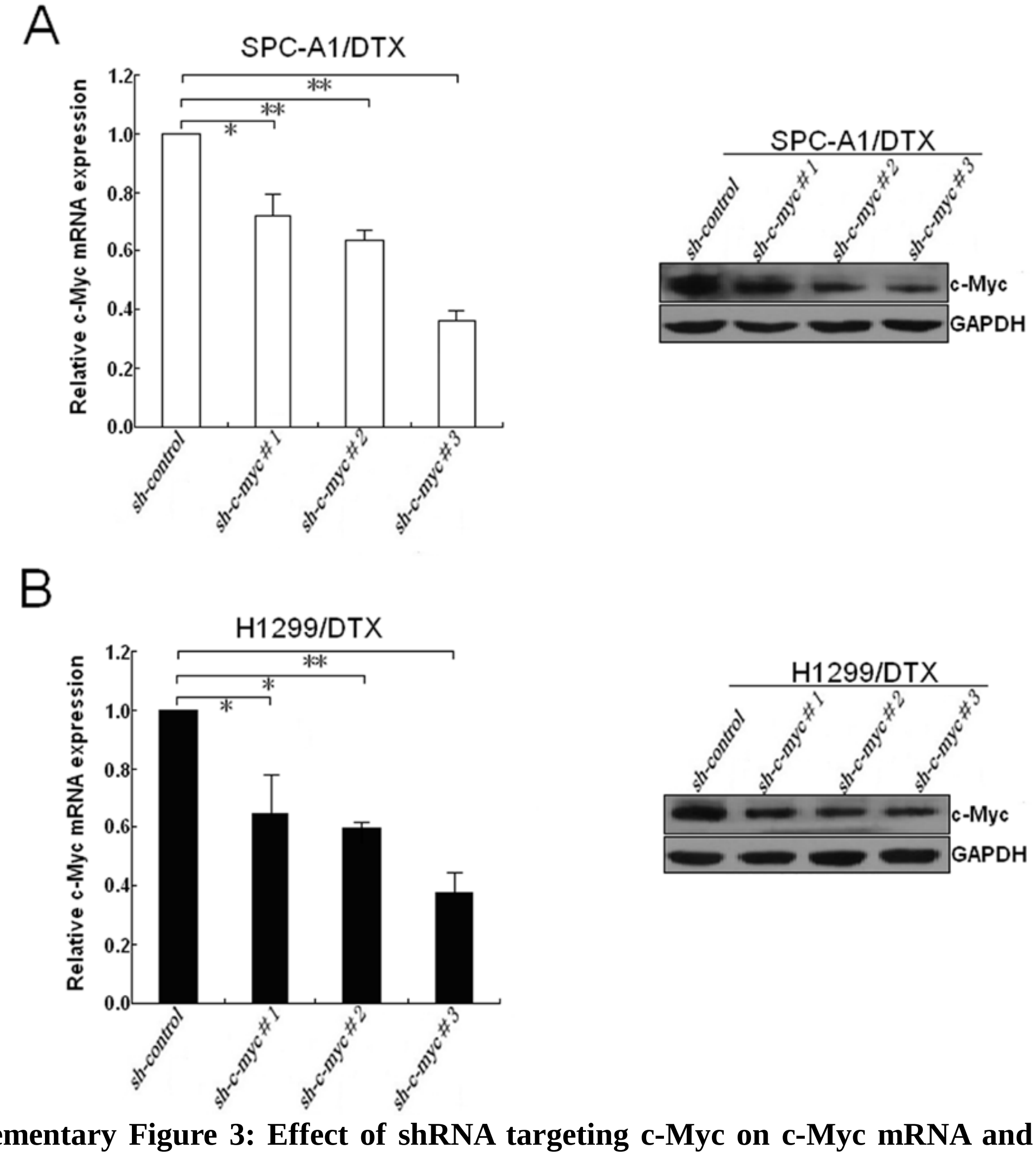

Supplementary Figure 3: Effect of shRNA targeting c-Myc on c-Myc mRNA and protein expression in docetaxel-resistant LAD cells. (A) qRT-PCR and Western blotting detection of c-Myc mRNA and protein expression in SPC-A1/DTX stably transfected with sh-control, sh-c-Myc#1, sh-c-Myc#2 or sh-cMy#3 vector, respectively. (B) qRT-PCR and Western blotting detection of c-Myc mRNA and protein expression in H1299/DTX cells stably transfected with sh-control, sh-c-Myc#1, sh-c-Myc#2 or sh-cMy#3 vector, respectively. GAPDH was used as an internal control. Results represent the average of three independent experiments (mean±SD). $^{*}P<$ 0.05 and $^{**}P< 0.01$.

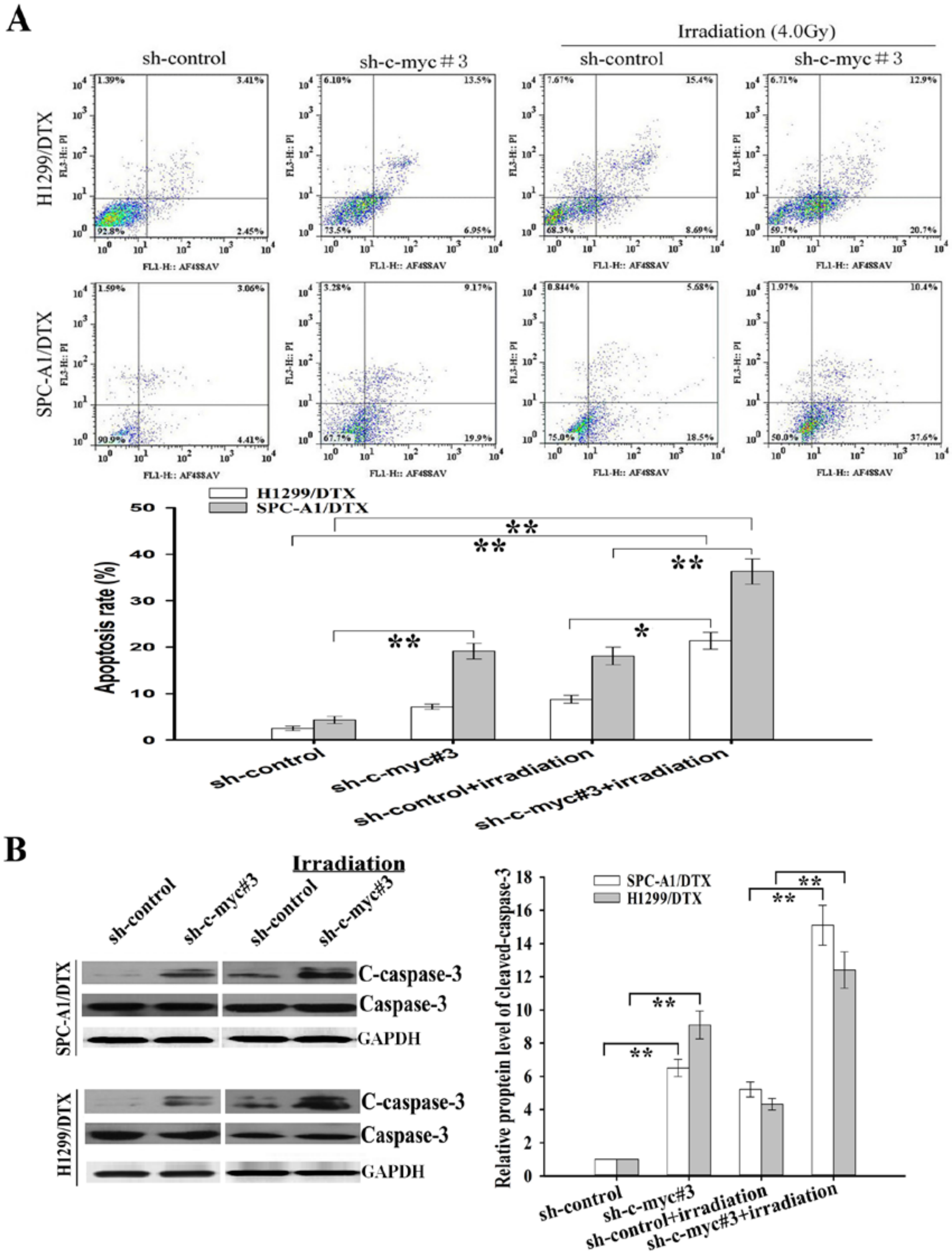

Supplementary Figure 4: Silencing of c-Myc increases irradiation-induced apoptosis of docetaxel-resistant LAD cells. (A) Flow cytometric analysis of apoptosis in sh-c-Myc#3 (or sh-control)-transfected H1299/DTX or SPC-A1/DTX cells treated without irradiation or with irradiation (4.0 Gy). (B) Western blotting detection of C-caspase-3 and Caspase-3 protein expression in sh-c-Myc#3 (or sh-control)-transfected H1299/DTX or SPC-A1/DTX cells treated without irradiation or with irradiation (4.0 Gy). GAPDH was used as an internal control. Results represent the average of three independent experiments (mean±SD). $^{*}P< 0.05$ and $^{**}P< 0.01$.

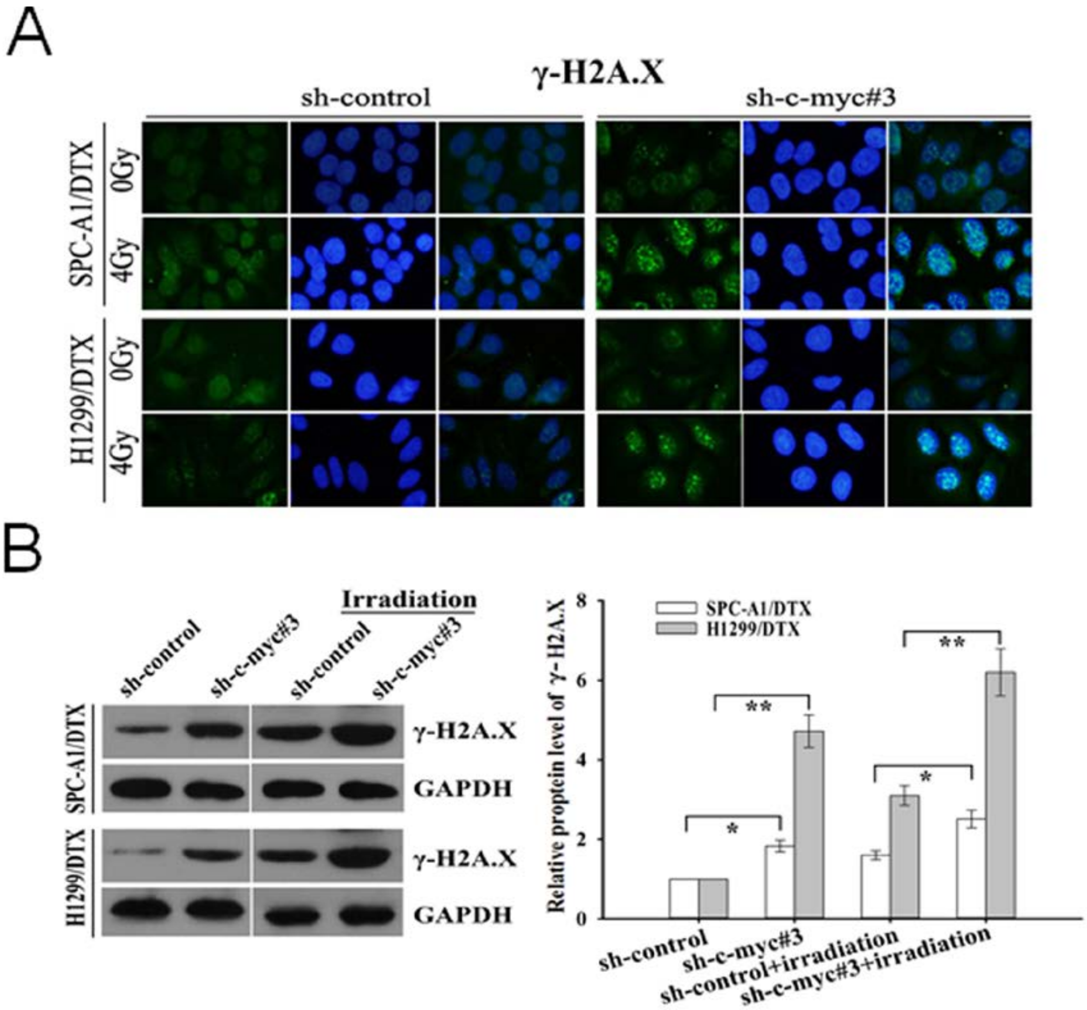

Supplementary Figure 5: Silencing of c-Myc increases irradiation-mediated DNA double-strand breaks (DSBs) of docetaxel-resistant LAD cells. (A) Immunofluorescence detection of phosphorylation of H2A.X (γ-H2A.X) foci formation (a marker of DSB) in sh-c-Myc#3 (or sh-control)-transfected SPC-A1/DTX or H1299/DTX treated without irradiation or with irradiation (4.0 Gy). (B) Western blotting detection of γ-H2A.X protein expression in sh-c-Myc#3 (or sh-control)-transfected SPC-A1/DTX or H1299/DTX treated without irradiation or with irradiation (4.0 Gy). GAPDH was used as an internal control. Results represent the average of three independent experiments (mean ± SD). $^{*}P < 0.05$ and $^{**}P < 0.01$.

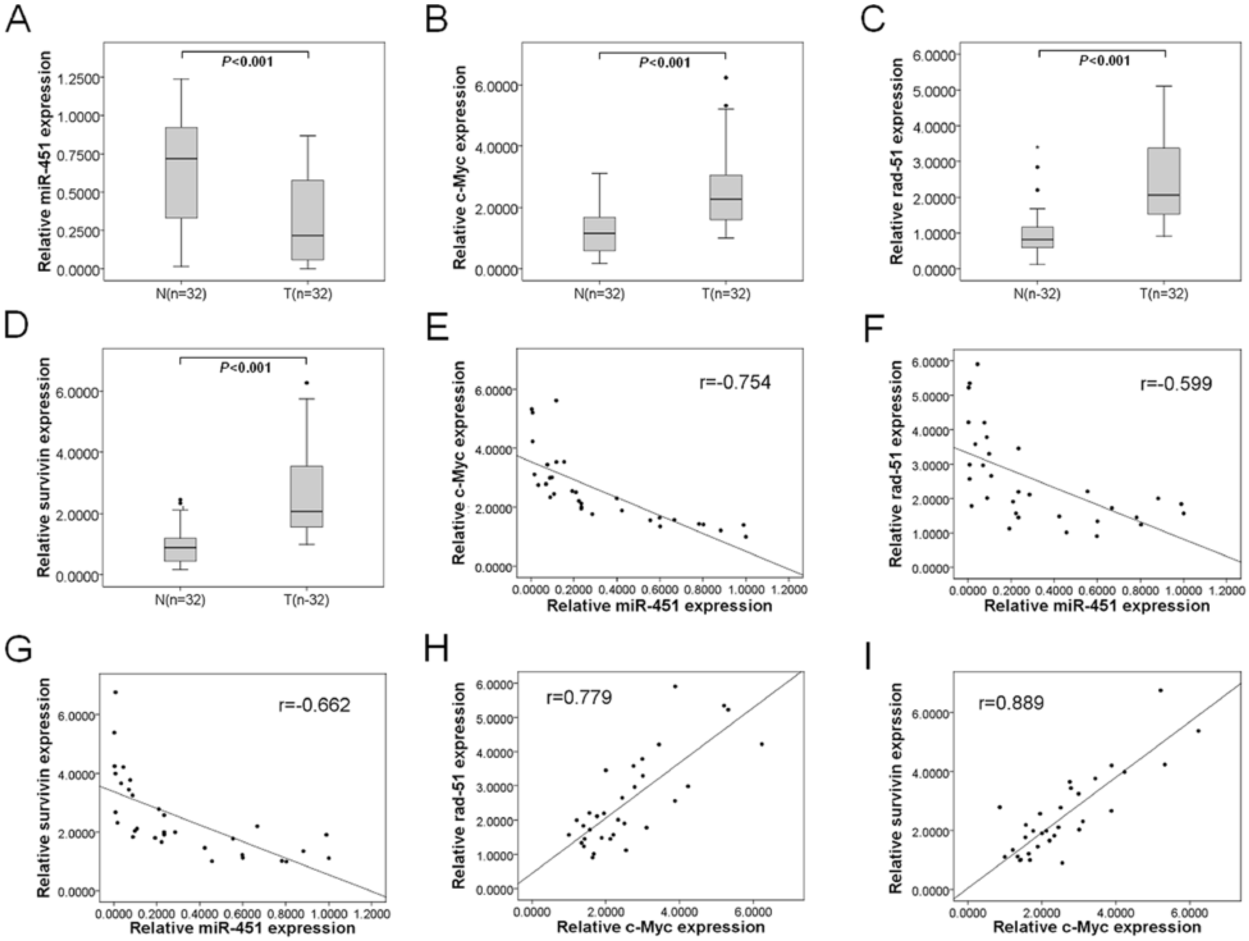

Supplementary Figure 6: Expression of miR-451, c-Myc, rad-51 and survivin mRNA in LAD tissues. (A) qRT-PCR detection of relative miR-451 expression in LAD tissues (n=32) and corresponding nontumor tissues (n=32; P<0.001). U6 was used as an internal control. (B) qRT-PCR detection of relative c-Myc mRNA expression in LAD tissues (n=32) and corresponding nontumor tissues (n=32; P<0.001). (C) qRT-PCR detection of relative rad-51 mRNA expression in LAD tissues (n=32) and corresponding nontumor tissues (n=32; P<0.001). (D) qRT-PCR detection of relative survivin mRNA expression in LAD tissues (n=32) and corresponding nontumor tissues (n=32; P<0.001). (E) A statistically significant inverse correlation between miR-451 and c-Myc mRNA expression levels in 32 cases of LAD tissues (Spearman's correlation analysis, r = -0.754; P<0.0001). (F) A statistically significant inverse correlation between miR-451 and rad-51 mRNA expression levels in 32 cases of LAD tissues (Spearman's correlation analysis, r = -0.599; P<0.01). (G) A statistically significant inverse correlation between miR-451 and survivin mRNA expression levels in 32 cases of LAD tissues (Spearman's correlation analysis, r = -0.599; P<0.001). (H) A statistically significant positive correlation between c-Myc and rad-51 mRNA expression levels in 32 cases of LAD tissues (Spearman's correlation analysis, r=0.779; P<0.001). (I) A statistically significant positive correlation between c-Myc and survivin mRNA expression levels in 32 cases of LAD tissues (Spearman's correlation analysis, r=0.889; P<0.001). Results represent the average of three independent experiments (mean±SD). Corresponding P values analyzed by Spearman correlation test are indicated.

Supplementary Table 1: Primers for qRT-PCR assay

Name	Primes
**miR-451**	**F:**5’-ACACTCCAGCTGGGAAACCGTTACCATTA -3’
	**R:**5’-TGGTGTCGTGGAGTCG-3’
**U6**	**F:**5’-CTCGCTTCGGCAGCACA-3’
	**R:**5’-AACGCTTCACGAATTTGCGT-3’
**c-Myc**	**F:**5’-GGAGGCTATTCTGCCCATTT-3’
	**R:**5’-CGAGGTCATAGTTCCTGTTGGT-3’
**Rad-51**	**F:**5’-CGCCCTTTACAGAACAGACTACT-3’
	**R:**5’-AAACATCGCTGCTCCATCC-3’
**Survivin**	**F:**5’-CGAGGCTGGCTTCATCCA-3’
	**R:**5’-GCAACCGGACGAATGCTTT-3’
**GAPDH**	**F:**5’-TGGGTGTGAACCATGAGAAGT-3’
	**R:**5’-TGAGTCCTTCCACGATACCAA-3’

Supplementary Table 2: The sequences of shRNAs and primers

Name	Primes
**sh-control**	**F** 5’-CCGGGCTTCTCCGAACGTGTCACGTCTCGAGAAGAAACCAGTAAACGTAAGCTTTTTG -3’
	**R** 5’- AATTCAAAAAGCTTCTCCGAACGTGTCACGTCTCGAGAAGAAACCAGTAAACGTAAGC -3’
**sh-c-myc #1**	**F** 5’- CCGGCCCAAGGTAGTTATCCTTAAACTCGAGTTTAAGGATAACTACCTTGggTTTTTG-3’
	**R**5’- AATTCAAAAACCCAAGGTAGTTATCCTTAAACTCGAGTTTAAGGATAACTACCTTGGG-3’
**sh-c-myc #2**	**F** 5’- CCGGCAGTTGAAACACAAACTTGAACTCGAGTTCAAGTTTGTGTTTCAACtgTTTTTG-3’
	**R** 5’- AATTCAAAAACAGTTGAAACACAAACTTGAACTCGAGTTCAAGTTTGTGTTTCAACTG-3’
**sh-c-myc #3**	**F** 5’- CCGGCAGGAACTATGACCTCGACTACTCGAGTAGTCGAGGTCATAGTTCCtgTTTTTG-3’
	**R** 5’- AATTCAAAAACAGGAACTATGACCTCGACTACTCGAGTAGTCGAGGTCATAGTTCCTG-3’
**pGL3/c-myc/3’-UTR-wt,**	
	**F:**5’-CATCTAGAGGAAAAGTAAGGAAAACGATTCCTTCT-3’,
	**R:**5’-GCTCTAGATATTAAAGTTATTTACATTTAATGGCA-3’
**pGL3/c-myc/3’-UTR-mut,**	
	**F:**5’-CATCTAGAGGAAAAGTAAGGAATCGCCGGCCTTCTAACA-3’
	**R:**5’-GCTCTAGATATTAAAGTTATTTACATTTAATGGCA-3’

Supplementary Table 3: Primers for CHIP assay

Name	Primes
**Rad-51**	**F:**5'-AGAGATGGGGTTTTGCCATC-3'
	**R:**5'-GTGGCTCAAGCCTGTAATCC-3'
**Survivin**	**F:**5'-CTGCACGCGTTCTTTGA-3'
	**R:**5'-GCGGTGGTCCTTGAG A-3'

Supplementary Table 4: Clinicopathological factors of patients

Variables	Number (%)
Gender	
Female	22 (68.8)
Male	10 (31.2)
Smoking condition	
Smoker	26 (81.2)
Non-smoker	6 (18.8)
Age (years)	
<65	18 (56.3)
≥65	14 (43.7)
Tumor differentiation	
Well+Moderate	11 (34.3)
Poor	21 (65.7)
Clinical stage	
IIIB	19 (59.4)
IV	13 (40.6)
Tumor response	
CR+PR	8 (25.0)
SD+PD	24 (75.0)
